# The Moral Consideration of Artificial Entities: A Literature Review

**DOI:** 10.1007/s11948-021-00331-8

**Published:** 2021-08-09

**Authors:** Jamie Harris, Jacy Reese Anthis

**Affiliations:** 1 Sentience Institute, New York, USA; 2 Department of Sociology, University of Chicago, 1126 East 59th Street, Chicago, IL 60637 USA

**Keywords:** Artificial intelligence, Robots, Rights, Moral consideration, Ethics, Philosophy of technology

## Abstract

Ethicists, policy-makers, and the general public have questioned whether artificial entities such as robots warrant rights or other forms of moral consideration. There is little synthesis of the research on this topic so far. We identify 294 relevant research or discussion items in our literature review of this topic. There is widespread agreement among scholars that some artificial entities could warrant moral consideration in the future, if not also the present. The reasoning varies, such as concern for the effects on artificial entities and concern for the effects on human society. Beyond the conventional consequentialist, deontological, and virtue ethicist ethical frameworks, some scholars encourage “information ethics” and “social-relational” approaches, though there are opportunities for more in-depth ethical research on the nuances of moral consideration of artificial entities. There is limited relevant empirical data collection, primarily in a few psychological studies on current moral and social attitudes of humans towards robots and other artificial entities. This suggests an important gap for psychological, sociological, economic, and organizational research on how artificial entities will be integrated into society and the factors that will determine how the interests of artificial entities are considered.

## Introduction

Recent decades have seen a substantial increase in human interaction with artificial entities. Robots manufacture goods (Shneier & Bostelman, 2015), care for the elderly (van Wynsberghe, 2013), and manage our homes (Young et al., 2009). Simulations are used for entertainment (Granic et al., 2014), military training (Cioppa et al., 2004), and scientific research (Terstappen & Reggiani, 2001). Further breakthroughs in artificial intelligence or space exploration may facilitate a vast proliferation of artificial entities (Reese, 2018; Baum et al., 2019; Anthis and Paez, 2021; Bostrom, 2003). Their increasing numbers and ubiquity raise an important question of moral consideration.

Policy-makers have begun to engage with this question. A 2006 paper commissioned by the U.K. Office of Science argued that robots could be granted rights within 50 years (BBC, 2006). South Korea proposed a “robot ethics charter” in 2007 (Yoon-mi, 2010). Paro, a type of care robot in the shape of a seal, was granted a “koseki” (household registry) in Nanto, Japan in 2010 (Robertson, 2014). The European Parliament passed a resolution in 2017 suggesting the creation of “a specific legal status for robots in the long run, so that at least the most sophisticated autonomous robots could be established as having the status of electronic persons” (European Parliament Committee on Legal Affairs, 2017). In the same year, a robot named Sophia was granted citizenship in Saudi Arabia (Hanson Robotics, 2018) and a chatbot on the messaging app Line, named Shibuya Mirai, was granted residence by the city of Tokyo in Japan (Microsoft Asia News Center, 2017).

Policy decisions relating to the rights of artificial entities have been reported in the media (Browne, 2017; Maza, 2017; Reynolds, 2018; Weller, 2020), discussed by the public,^1^ and critiqued by academics (Open Letter, 2018). The moral consideration of artificial entities has also been explored extensively in science fiction (McNally & Inayatullah, 1988, p. 128; Petersen, 2007, pp. 43–4; Robertson, 2014, p. 573–4; Inyashkin, 2016; Kaminska, 2016; Arnold & Gough, 2017; Gunkel, 2018a, pp. 13–8; Hallqvist, 2018; Kunnari, 2020). People for the Ethical Treatment of Reinforcement Learners have explicitly advocated for the moral consideration of artificial entities that can suffer (PETRL, 2015) and The American Society for the Prevention of Cruelty to Robots have done so for those that are “self-aware” (Anderson, 2015).

Scholars often conclude that artificial entities with the capacity for positive and negative experiences (i.e. sentience) will be created, or are at least theoretically possible (see, for example, Thompson, 1965; Aleksander, 1996; Buttazzo, 2001; Blackmore, 1999; Franklin, 2003; Harnad, 2003; Holland, 2007; Chrisley, 2008; Seth, 2009; Haikonen, 2012; Bringsjord et al., 2015; Reese, 2018; Anthis and Paez, 2021; Angel, 2019). Surveys of cognitive scientists (Francken et al., 2021) and artificial intelligence researchers (McDermott, 2007) suggest that many are open to this possibility. Tomasik (2011), Bostrom (2014), Gloor (2016a), and Sotala and Gloor (2017) argue that the insufficient moral consideration of sentient artificial entities, such as the subroutines or simulations run by a future superintelligent AI, could lead to astronomical amounts of suffering. Kelley and Atreides (2020) have already proposed a “laboratory process for the assessment and ethical treatment of Artificial General Intelligence systems that could be conscious and have subjective emotional experiences.”

There has been limited synthesis of relevant literature to date. Gunkel (2018a) provides the most thorough review to set up his argument about “robot rights,” categorizing contributions into four modalities: “Robots Cannot Have Rights; Robots Should Not Have Rights,” “Robots Can Have Rights; Robots Should Have Rights,” “Although Robots Can Have Rights, Robots Should Not Have Rights,” and “Even if Robots Cannot Have Rights, Robots Should Have Rights.” Gunkel critiques each of these perspectives, advocating instead for “thinking otherwise” via deconstruction of the questions of whether robots can and should have rights. Bennett and Daly (2020) more briefly summarize the literature on these two questions, adding a third: “*will* robots be granted rights?” They focus on legal rights, especially legal personhood and intellectual property rights. Tavani (2018) briefly reviews the usage of “robot” and “rights,” the criteria necessary for an entity to warrant moral consideration, and whether moral agency is a prerequisite for moral patiency, in order to explain a new argument that social robots warrant moral consideration.

However, those reviews have not used systematic methods to comprehensively identify relevant publications or quantitative methods of analysis, making it difficult to extract general trends and themes.^2^ Do scholars tend to believe that artificial entities warrant moral consideration? Are views split along geographical and disciplinary lines? Which nations, disciplines, and journals most frequently provide contributions to the discussion? Using a systematic search methodology, we address these questions, provide an overview of the literature, and suggest opportunities for further research. Common in social science and clinical research (see, for example, Higgins and Green, 2008; Campbell Collaboration, 2014), systematic reviews have recently been used in philosophy and ethics research (Nill & Schibrowsky, 2007; Mittelstadt, 2017; Hess and Fore, 2017; Saltz & Dewar, 2019; Yi et al., 2019).

Previous reviews have also tended to focus on “robot rights.” Our review has a broader scope. We use the term “artificial entities” to refer to all manner of machines, computers, artificial intelligences, simulations, software, and robots created by humans or other entities. We use the phrase “moral consideration” of artificial entities to collectively refer to a number of partly overlapping discussions: whether artificial entities are “moral patients,” deserve to be included in humanity’s moral circle, should be granted “rights,” or should otherwise be granted consideration. Moral consideration does not necessarily imply the attribution of intrinsic moral value. While not the most common,^3^ these terms were chosen for their breadth.

## Methodology

Four scientific databases (Scopus, Web of Science, ScienceDirect, and the ACM Digital Library) were searched systematically for relevant items in August and September 2020. Google Scholar was also searched, since this search engine is sometimes more comprehensive, particularly in finding the grey literature that is essential to cataloguing an emerging field (Martín-Martín et al., 2019).

Given that there is no single, established research field examining the moral consideration of artificial entities, multiple searches were conducted to identify relevant items; a total of 2692 non-unique items were screened for inclusion (see Table 1). After exclusions (see criteria below) and removal of duplicates, 294 relevant research or discussion items were included (see Table 2; see the “Appendix” for item summaries and analysis).

**Table 1:** Initial results returned for screening, by search terms and search location

Search category	Search term	Scopus	Web of Science	Science Direct	ACM Digital Library	Google Scholar
“rights”	“robot rights”	42	22	13	29	939
	“rights for robots”	4	3	33	7	179
	“machine rights” OR “rights for machines”^a^	35	6	36	3	267
	“artificial intelligence rights” OR “rights for artificial intelligence”	2	1	13	0	59
“moral”	(“moral consideration” OR “moral concern”) AND (robots OR machines OR “artificial intelligence”)	54	12	529	102	8690
	(“moral circle” OR “moral expansiveness”) AND (robots OR machines OR “artificial intelligence”)	4	2	13	5	420
	(“Moral patient” OR “moral patients” OR “moral patiency”) AND (robots OR machines OR “artificial intelligence”)	25	11	28	52	1290
“suffering”	“suffering subroutines”	0	0	0	0	18
	(“mind crime” OR “mindcrime”) AND simulations	0	0	0	2	82
	(“astronomical suffering” OR “suffering risks”)	11	5	46	4	277

^a^The Google Scholar search for “Machine rights” OR “rights for machines” included the additional operator -Kathrani, because the search otherwise returned a large number of webpages that all referred back to a single talk

**Table 2:** Included items, by search terms and search location

Search category	Search term	Scopus	Web of science	Science direct	ACM digital library	Google Scholar
“rights”	“robot rights”	20	13	4	18	70
	“rights for robots”	2	1	6	4	50
	“machine rights” OR “rights for machines”	6	5	2	1	23
	“artificial intelligence rights” OR “rights for artificial intelligence”	2	1	2	0	9
“moral”	(“moral consideration” OR “moral concern”) AND (robots OR machines OR “artificial intelligence”)	20	9	6	40	82
	(“moral circle” OR “moral expansiveness”) AND (robots OR machines OR “artificial intelligence”)	2	0	5	2	27
	(“Moral patient” OR “moral patients” OR “moral patiency”) AND (robots OR machines OR “artificial intelligence”)	14	7	4	27	75
“suffering”	“suffering subroutines”	0	0	0	0	9
	(“mind crime” OR “mindcrime”) AND simulations	0	0	0	1	14
	(“astronomical suffering” OR “suffering risks”)	2	2	1	0	8

For the database searches, the titles and abstracts of returned items were reviewed to determine relevance. For the Google Scholar searches, given the low relevance of some returned results, review was limited to the first 200 results, similar to the approach of Mittelstadt (2017).

Common reasons for exclusion were that the item:

- Did not discuss the moral consideration of artificial entities (e.g. discussed whether artificial entities could be moral agents but not whether they could be moral patients^4^),
- Mentioned the topic only very briefly (e.g. only as thought-provoking issue adjacent to the main focus of the article), or
- Were not in the format of an academic article, book, conference paper, or peer-reviewed magazine contribution (e.g. they were published as a newspaper op-ed or blog post^5^).

The findings are analyzed qualitatively and discussed in the sections below. Results are also categorized and scored along the following dimensions:

- Categories of the search terms that identified each item, which reflect the language used by the authors; the three categories used are “rights,” “moral,” and “suffering” searches,
- Categories of academic disciplines of the lead author of each included item,
- Categories of primary frameworks or moral schemas used, similar to the approach of Hess and Fore (2017), and
- A score representing the author’s position on granting moral consideration to artificial entities on a scale from 1 (argues forcefully against consideration, e.g. suggesting that artificial beings should never be considered morally) to 5 (argues forcefully for consideration, e.g. suggesting that artificial beings deserve moral consideration now).

In addition to the discussion below, the “Appendix” includes a summary of each item and the full results of the categorization and scoring analyses.

## Results

### Descriptive Statistics

Included items were published in 106 different journals. Four journals published more than five of the included items; *Ethics and Information Technology* (9% of items), *AI and Society* (4%), *Philosophy and Technology* (2%), and *Science and Engineering Ethics* (2%). Additionally, 15% of items were books or chapters (only one book focused solely on this topic was identified, Gunkel, 2018a),^6^ 13% were entries in a report of a conference, workshop, or symposium (often hosted by the Association for Computing Machinery or Institute of Electrical and Electronics Engineers), and 12% were not published in any journal, magazine, or book.

The included items were produced by researchers affiliated with institutions based in 43 countries. Only five countries produced more than 10 of the identified items: the United States (36% of identified items), the United Kingdom (15%), the Netherlands (7%), Australia (5%), and Germany (4%). According to Google Scholar, included items have been cited 5992 times (excluding one outlier with 2513 citations, Bostrom, 2014); 41% of these citations are of items produced in the US.^7^

**Fig. 1:**
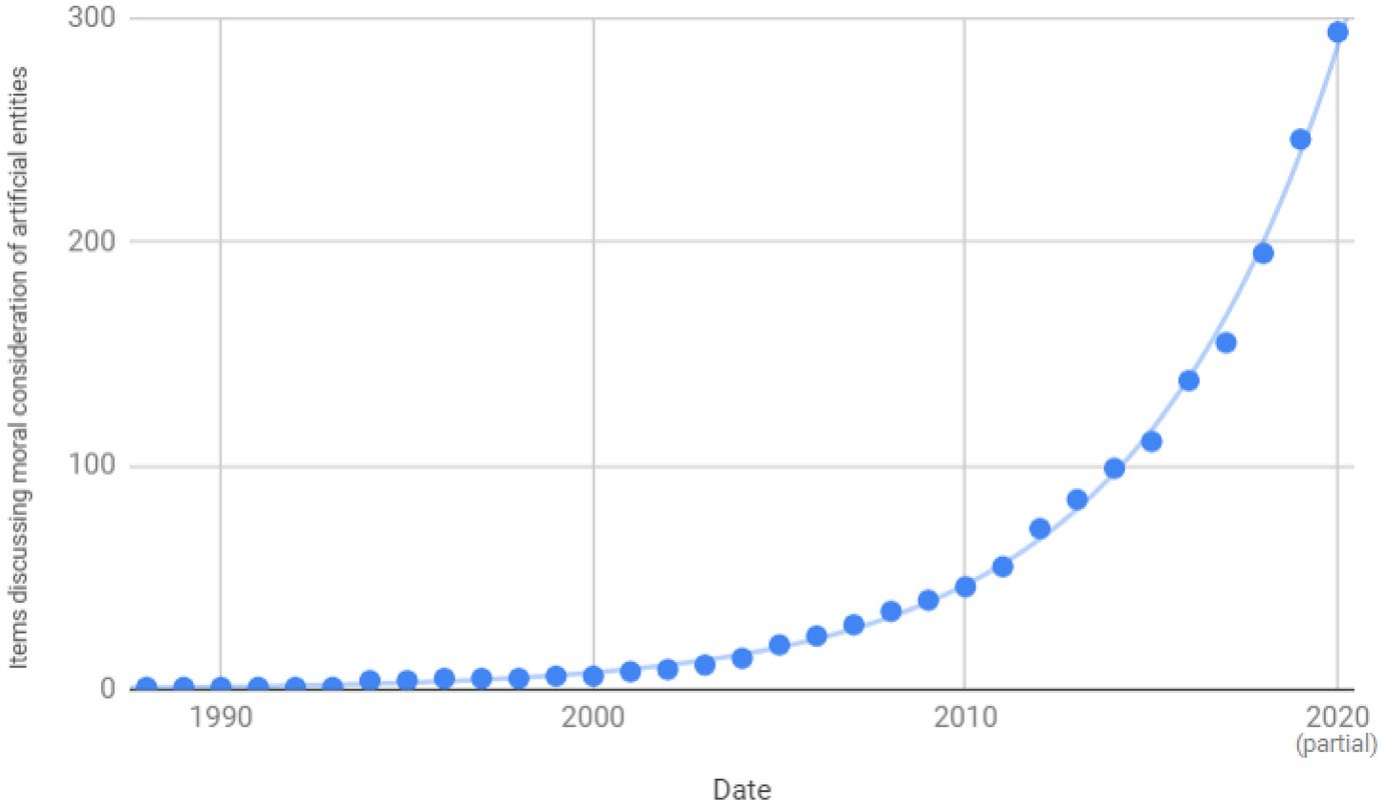
Cumulative total of included items, by date of publication

The oldest included item identified by the searches was McNally and Inayatullah (1988), though included items cited articles from as early as 1964 as offering relevant comments (Freitas, 1985; Lehman-Wilzig, 1981; Putman, 1964; Stone, 1974). The study of robot ethics (now called “roboethics” by some (Veruggio & Abney, 2012, pp. 347–8)) grew in the early 2000s (Malle, 2016). Levy (2009), Torrance (2013, p. 403), and Gunkel (2018c, p. 87) describe the moral consideration of artificial entities as a small and neglected sub-field. However, the results of this literature review suggest that academic interest in the moral consideration of artificial entities is growing exponentially (see Fig. 1).

As shown in Table 3, the most common academic disciplines of contributing scholars are philosophy or ethics, law, computer engineering or computer science, and communication or media. We focus on the primary disciplines of scholars, rather than of publications, because so many of the publications are interdisciplinary.

**Table 3:** Items and citations by the academic discipline of the lead author

Academic discipline	Count and citations		Percentages of total	
	Count of items	Citation count (outlier excluded)^a^	Count of items	Citation count (outlier excluded)
Philosophy or ethics	82	2101	28	35
Law	49	1002	17	17
Computer engineering or computer science	30	473	10	8
Communication or media	28	740	10	12
Psychology	20	495	7	8
Other social sciences	18	111	6	2
Other engineering	12	200	4	3
Cognitive science	10	300	3	5
Other humanities	8	14	3	0
Medical or biology	6	111	2	2
Information technology	5	86	2	1
Design	5	27	2	0
Robotics	4	23	1	0
History	3	83	1	1
Business	3	17	1	0
Political science	3	13	1	0
English literature or language	3	0	1	0
Future studies	2	3	1	0
Other or unidentifiable	32	471	11	8
Total	294	5992	100	100

^a^The outlier is Bostrom (2014). If this item is included, then the citation count for philosophy or ethics rises to 4614, which is 54% of the total

As shown in Table 4, many scholars contributing to the discussion do not adopt a single, clear moral schema, focusing instead on legal precedent, empirical evidence of attitudes towards artificial entities, or simply summarizing the views of previous scholars (e.g. Weng et al., 2009, p. 267; Gray & Wegner, 2012, pp. 125–30).

**Table 4:** Items and citations by the primary framework or moral schema used

Primary framework or moral schema	Count and citations		Percentages of total	
	Count of items	Citation count (outlier excluded)^a^	Count of items	Citation count (outlier excluded)
Legal precedent	34	906	12	15
Social-relational	23	606	8	10
Consequentialist	16	99	5	2
Deontological	15	213	5	4
Information ethics	5	1019	2	17
Virtue ethicist	5	28	2	0
Not Applicable	103	1641	35	27
Mixture	52	892	18	15
Unclear	26	277	9	5
Other	15	311	5	5
Total	294	5992	100	100

^a^The outlier is Bostrom (2014). If this item is included, then the citation count for the consequentialist category rises to 2612, which is 31% of the total

Many scholars use consequentialist, deontological, or virtue ethicist moral frameworks, or a mixture of these. These scholars defend various criteria as crucial for determining whether artificial entities warrant moral consideration. Sentience or consciousness seem to be most frequently invoked (Andreotta, 2020; Bostrom, 2014; Himma, 2003; Johnson & Verdicchio, 2018; Mackenzie, 2014; Mosakas, 2020; Tomasik, 2014; Torrance, 2008; Yampolskiy, 2017), but other proposed criteria include the capacities for interests (Basl, 2014; Neely, 2014), autonomy (Calverley, 2011; Gualeni, 2020), self-control (Wareham, 2013), rationality (Laukyte, 2017), integrity (Gualeni, 2020), dignity (Bess, 2018), moral reasoning (Malle, 2016), and virtue (Gamez et al., 2020).

Some of the most influential scholars propose more novel ethical frameworks. Coeckelbergh (2010a, 2010b, 2014, 2018, 2020) and Gunkel (2013, 2014, 2015, 2018a, 2018b, 2018c, 2018d, 2019a, 2019b, 2020b), encourage a social-relational framework to discuss the moral consideration of artificial entities. This approach grants moral consideration on the basis of how an entity “is treated in actual social situations and circumstances” (Gunkel, 2018a, p. 10). Floridi (1999, 2002, 2005) encourages “information ethics,” where “[a]ll entities, qua informational objects, have an intrinsic moral value.” Though less widely cited, Danaher’s (2020) theory of “ethical behaviorism” and Tavani’s (2018) discussion of “being-in-the-technological-world” arguably offer alternative moral frameworks for assessing whether artificial entities warrant moral consideration. Non-Western frameworks also differ in their implications for the moral consideration of artificial entities (Gunkel, 2020a; McNally & Inayatullah, 1988).

### Focus and Terminology

Definitions of the widely-used term “robot” are varied and often vague (Lin et al., 2011, pp. 943–4; Robertson, 2014, p. 574; Tavani, 2018, pp. 2–3; Gunkel, 2018a, pp. 14–26; Beno, 2019, pp. 2–3). It can be defined broadly, such as “a machine that resembles a living creature in being capable of moving independently (as by walking or rolling on wheels) and performing complex actions (such as grasping and moving objects)” (Merriam-Webster, 2008). More narrowly, to many people, the term robot implies a humanoid appearance, or at least humanoid functions and behaviors (Brey & Søraker, 2009; Leenes & Lucivero, 2014; Rademeyer, 2017). This terminology seems suboptimal, given that the forms of artificial sentience that seem most at risk of experiencing intense suffering on a large scale in the long-term future may not have humanoid characteristics or behaviors; they may even exist entirely within computers, not having any embodied form, human or otherwise.^8^ Other terms used by scholars include “artificial beings” (Gualeni, 2020), “artificial consciousness” (Basl, 2013b), “artificial entities” (Gunkel, 2015), “artificial intelligence” (Ashrafian, 2015b), “artificial life” (Sullins, 2005), “artificial minds” (Jackson Jr, 2018a), “artificial person” (Michalski, 2018), “artificial sentience” (Ziesche & Yampolskiy, 2019), “machines” (Church, 2019), “automata” (Miller, 2015), computers (Drozdek, 1994), “simulations” (Bostrom, 2014), and “subroutines” (Winsby, 2013). Alternative adjectives such as “synthetic,” “electronic,” and “digital” are also sometimes used to replace “artificial.”^9^

Relevant discussion has often focused on the potential “rights” of artificial entities (Tavani, 2018, pp. 2–7; Gunkel, 2018a, pp. 26–33). There has been some debate over whether “rights” is the most appropriate term, given its ambiguity and that legal and moral rights are each only one mechanism for moral consideration (Kim & Petrina, 2006, p. 87; Tavani, 2018, pp. 4–5; Cappuccio et al., 2020, p. 4). Other scholars consider whether artificial entities can be “moral patients,” granted “moral consideration,” or included in the “moral circle” (Cappuccio et al., 2020; Danaher, 2020; Küster & Świderska, 2016). Some scholars use terminology that focuses on the suffering of specific forms of artificial sentience: “mind crime” against simulations (Bostrom, 2014), “suffering subroutines” (Tomasik, 2011), or “risks of astronomical future suffering” (Tomasik, 2011) and the derivative term “s-risks.”

There were more items found by “rights” or “moral” than “suffering” search terms (see Table 5). Although 31% of the items identified by “rights” search terms were also identified by “moral” search terms, only 12% of the results from the “suffering” search terms were also identified by “rights” or “moral” search terms. Additionally, excluding one outlier—Bostrom (2014)—items identified via the “suffering” search terms had a lower average citation count (8) than items identified via “moral” (24) or “rights” (20) search terms. If the outlier is included, then the average for the suffering search terms is over ten times larger (108), and these items comprise 32% of the total citations (see “Appendix”).

**Table 5:** Items and citations by search term category

Search term category	Count and citations		Percentages of total	
	Count of items	Citation count (outlier excluded)	Count of items	Citation count (outlier excluded)
“Rights” search terms	146	2938	50	49
“Moral” search terms	171	4071	58	68
“Suffering” search terms	25	187	9	3
Total	294	5992	100	100

The terminology used varied by the authors’ academic discipline and moral framework. For example, the items by legal scholars were mostly identified by “rights” search terms (80%) while the items by psychologists were mostly identified by “moral” search terms (90%). In the “other or unidentifiable” category, 44% were identified via “suffering” search terms; these contributions were often by the Center on Long-Term Risk and other researchers associated with the effective altruism community.^10^ An unusually high proportion of “consequentialist” items were identified by “suffering” search terms (50%). None of the “information ethics” items were identified via “rights” search terms, whereas an unusually high proportion of the “legal precedent” items were identified this way (94%).

The primary questions that are addressed in the identified literature are (1) *Can or could* artificial entities ever be granted moral consideration? (2) *Should* artificial entities be granted moral consideration?^11^ The authors use philosophical arguments, ethical arguments, and arguments from legal precedent. They sometimes motivate their arguments with concern for the artificial entities themselves, but others argue in favor of the moral consideration of artificial entities because of positive indirect effects on human society, particularly on moral character (Levy, 2009; Davies, 2011; Darling, 2016, p. 215). Others argue against the moral consideration of artificial entities because of potentially damaging effects on human society (Bryson, 2018; Gerdes, 2016). Some items, especially those identified via the “moral” search terms, focus on a third question, (3) *What* attitudes do humans currently have vis-a-vis artificial entities, and what predicts these attitudes?^12^ A small number of contributions, especially those identified via the “suffering” search terms, also explicitly discuss (4) *What* are the best approaches to ensuring that the suffering of artificial sentience is minimized or that other interests of artificial entities are protected (e.g. Ashrafian, 2015a; Gloor, 2016b)? Others ask (5) *Should* humanity avoid creating machines that are complex or intelligent enough that they warrant moral consideration (e.g. Basl, 2013a; Beckers, 2018; Bryson, 2018; Hanák, 2019; Johnson & Verdicchio, 2018; McLaughlin & Rose, 2018; Tomasik, 2013)?

### Dismissal of the Importance of Moral Consideration of Artificial Entities

Calverley’s (2011) chapter in a book on *Machine Ethics* opens with the statement that, “[t]o some, the question of whether legal rights should, or even can, be given to machines is absurd on its face. How, they ask, can pieces of metal, silicon, and plastic, have any attributes that would allow society to assign it any rights at all.” Referring to his 1988 essay with Phil McNally, Sohail Inayatullah (2001) notes that he received substantial criticism from colleagues for writing about the topic of robot rights: “Pakistani colleagues have mocked me saying that Inayatullah is worried about robot rights while we have neither human rights, economic rights or rights to our own language and local culture… Others have refused to enter in collegial discussions on the future with me as they have been concerned that I will once again bring up the trivial.”

Some scholars dismiss discussion of the moral consideration of artificial entities as premature or frivolous, a distraction from concerns that they view as more pressing, usually concerns about the near-term consequences of developments in narrow artificial intelligence and social robots. For example, Birhane and van Dijk (2020) argue that, “the ‘robot rights’ debate is focused on first world problems, at the expense of urgent ethical concerns, such as machine bias, machine elicited human labour exploitation, and erosion of privacy all impacting society’s least privileged individuals.” Cappuccio et al. (2020, p. 3) suggest that arguments in favor of moral consideration for artificial entities that refer to “objective qualities or features, such as freedom of will or sentience” are “problematic because existing social robots are too unsophisticated to be considered sentient”; robots “do not display—and will hardly acquire any time soon—any of the objective cognitive prerequisites that could possibly identify them as persons or moral patients (e.g., self-awareness, autonomous decision, motivations, preferences).” This resembles critiques offered by Coeckelbergh (2010b) and Gunkel (2018c). McLaughlin and Rose (2018) refer to such “objective qualities” but note that, “[r]obot-rights seem not to be much of an issue” in roboethics because “the robots in question will be neither sentient nor genuinely intelligent… for the foreseeable future.”

Gunkel (2018a, pp. 33–44) provides a number of other examples of critics arguing that discussion of the moral consideration of artificial entities is “ridiculous,” as well as cases where it is “given some brief attention only to be bracketed or carefully excluded as an area that shall not be given further thought” or “included by being pushed to the margins of proper consideration.”

Despite these attitudes, our analysis shows that academic discussion of the moral consideration of artificial entities is increasing (see Fig. 1). This provides evidence that many scholars believe this topic is worth addressing. Indeed, Ziesche and Yampolskiy (2018, p. 2) have proposed the development and formalization of a field of “AI welfare science.” They suggest that, “[t]he research should target both aspects for sentient digital minds not to suffer anymore, but also for sentient and non-sentient digital minds not to cause suffering of other sentient digital minds anymore.”

Moreover, these dismissals do not engage with the long-term moral risks discussed in items identified via the “suffering” search terms. Wright (2019) briefly considers the “longer-term” consequences of granting “constitutional rights” to “advanced robots,” noting that doing so might spread resources thinly, but this is one of the only items not identified by the “suffering” search terms that explicitly considers the long-term future.^13^

### Attitudes Towards the Moral Consideration of Artificial Entities Among Contributing Scholars

We might expect different moral frameworks to have radically different implications for attitudes towards the appropriate treatment of artificial entities. Even where scholars share similar moral frameworks, their overall attitudes sometimes differ due to varying timeframes of evaluation or estimations of the likelihood that artificial entities will develop relevant capacities, among other reasons. For example, many scholars use sentience or consciousness as the key criterion determining whether an artificial entity is worthy of moral consideration, and most of these scholars remain open to the possibility that these entities will indeed become sentient in the future. Bryson et al. (2017) view consciousness as an important criterion but note that, “there is no guarantee or necessity that AI [consciousness] will be developed.”

The average consideration score (on a scale of 1 to 5) was 3.8 (standard deviation of 0.86) across the 192 items for which a score was assigned, indicating widespread, albeit not universal, agreement among scholars that at least some artificial entities could warrant moral consideration in the future, if not also the present. Where there is enough data to make meaningful comparisons, there is not much difference in average consideration score by country, academic discipline, or the primary framework or moral schema used (see “Appendix”).

However, our search terms will have captured only those scholars who deem the subject worthy of at least a passing mention. Other scholars interested in roboethics who consider the subject so “absurd,” “ridiculous,” “fanciful,” or simply irrelevant to their own work that they do not refer to the relevant literature will not have been identified. Bryson’s (2010) article “Robots Should be Slaves,” which argues against the moral consideration of current robots and against creating robots that can suffer, though cited 183 times, was not identified by the searches conducted here because of the terminology used in the article.

Individuals in disciplines associated with technical research on AI and robotics may be, on average, more hostile to granting moral consideration to artificial entities than researchers from other disciplines. We found that computer engineers and computer scientists had a lower average consideration score than other disciplines (2.6). Additionally, there are many roboticist and AI researcher signatories of the “Open Letter to the European Commission Artificial Intelligence and Robotics” (2018), which objects to a proposal of legal personhood for artificial entities, and when discussion of robot rights has gained media attention, many of the vocal critics appear to have been associated with computer engineering or robotics (Randerson, 2007; Yoon-mi, 2010; Gunkel, 2018a, pp. 35–6). Relatedly, Zhang and Dafoe (2019) found in their US survey that respondents with computer science or engineering degrees “rate all AI governance challenges as less important” than other respondents. In this sense, resistance to the moral consideration of artificial entities may fall under a general category of “AI governance” or “AI ethics,” which technical researchers may see as less important than other stakeholders. These technical researchers may not disagree with the proponents of moral consideration of artificial entities; they may simply have a different focus, such as incremental technological progress rather than theorizing about societal trajectories.

### Empirical Research on Attitudes Towards the Moral Consideration of Artificial Entities

Five papers (Hughes, 2005, Nakada, 2011, Nakada, 2012, Spence et al., 2018; Lima et al., 2020) included surveys testing whether individuals believe that artificial entities might plausibly warrant moral consideration in the future. Agreement with statements favorable to future moral consideration varied from 9.4 to 70%; given the variety of question wordings, participant nationalities, and sampling methods (students, online participants, or members of the World Transhumanist Association), general trends are difficult to extract.

There are a number of surveys and experiments on attitudes towards current artificial entities. Some of this research provides evidence that people empathize with artificial entities and respond negatively to actions that appear to harm or insult them (Darling, 2016; Freier, 2008; Rosenthal-von der Pütten et al., 2013; Suzuki et al., 2015). Bartneck and Keijsers (2020) found no significant difference between participants’ ratings of the moral acceptability of abuse towards a human or a robot, but other researchers have found evidence that current artificial entities are granted less moral consideration than humans (Slater et al., 2006; Gray et al., 2007; Bartneck & Hu, 2008; Küster & Świderska, 2016; Akechi et al., 2018; Sommer et al., 2019; Nijssen et al., 2019; Küster and Świderska, 2020).

Studies have found that people are more willing to grant artificial entities moral consideration when they have humanlike appearance (Küster et al., 2020; Nijssen et al., 2019), have high emotional (Nijssen et al., 2019; Lee et al., 2019) or mental capacities (Gray & Wegner, 2012; Nijssen et al., 2019; Piazza et al., 2014; Sommer et al., 2019), verbally respond to harm inflicted on them (Freier, 2008), or seem to act autonomously (Chernyak & Gary, 2016). There is also evidence that people in individual rather than group settings (Hall, 2005), with prior experience interacting with robots (Spence et al., 2018), or presented with information promoting support for robot rights, such as “examples of non-human entities that are currently granted legal personhood” (Lima et al., 2020) are more willing to grant artificial entities moral consideration. Other studies have examined the conditions under which people are most willing to attribute high mental capacities to artificial entities (Briggs et al., 2014; Fraune et al., 2017; Gray & Wegner, 2012; Küster & Swiderska, 2020; Küster et al., 2020; McLaughlin & Rose, 2018; Swiderska & Küster, 2018, 2020; Wallkötter et al., 2020; Wang & Krumhuber, 2018; Ward et al., 2013; Wortham, 2018).

## Limitations

Given that interest in this topic is growing exponentially, this review inevitably misses many recent relevant contributions. For example, a Google Scholar search for “robot rights” in July 2021 limited to 2021 returns 152 results, including a qualitative review (Gordon & Pasvenskiene, 2021). The chosen search terms likely miss some relevant items. They assume some level of abstraction to discuss “rights,” “moral,” or “suffering” issues explicitly; discussion which implicitly addresses these issues (e.g. Elder, 2017) may not have been included. This review’s exclusion criteria maintain coherence and concision but limit its scope. Future reviewers could adopt different foci, such as including discussion of the moral agency of artificial entities or contributions not using academic formats.

## Concluding Remarks

Many scholars lament that the moral consideration of artificial entities is discussed infrequently and not viewed as a proper object of academic inquiry. This literature review suggests that these perceptions are no longer entirely accurate. The number of publications is growing exponentially, and most scholars view artificial entities as potentially warranting moral consideration. Still, there are important gaps remaining, suggesting promising opportunities for further research, and the field remains small overall with only 294 items identified in this review.

These discussions have taken place largely separately from each other: legal rights, moral consideration, empirical research on human attitudes, and theoretical exploration of the risks of astronomical suffering among future artificial entities. Further contributions should seek to better integrate these discussions. The analytical frameworks used in one topic may offer valuable contributions to another. For example, what do legal precedent and empirical psychological research suggest are the most likely outcomes for future artificial sentience (as an example of studying likely technological outcomes, see Reese and Mohorčich, 2019)? What do virtue ethics and rights theories suggest is desirable in these plausible future scenarios?

Despite interest in the topic from policy-makers and the public, there is a notable lack of empirical data about attitudes towards the moral consideration of artificial entities. This leaves scope for surveys and focus groups on a far wider range of predictors of attitudes, experiments that test the effect of various messages and content on these attitudes, and qualitative and computational text analysis of news articles, opinion pieces, and science fiction books and films that touch on these topics. There are also many theoretically interesting questions to be asked about how these attitudes relate to other facets of human society, such as human in-group-out-group and human-animal interactions.

## Data Availability

The datasets generated during and/or analyzed during the current study are available in “Appendix”.

## Appendix

See Tables 6, 7, 8, 9, 10, 11 and 12.

**Table 6:** Search results summaries

References	Summary
Abdullah (2018)	Abdullah sees robot rights that also protect human interests as “legal necessity” and as “in line with the objectives of Islamic Law.” Abdullah comments that, “the criterion of bearing rights and duties in Islamic law,” which is known as “ahliyyah,” would not apply to robots. Liability for their actions would be “ascribed to the owner or manufacturer of the robot,” though this could change with the technology
Adam (2008)	Adam summarizes “the ways that Information Ethics (IE),” developed by Floridi, “treats things.” Most of the discussion focuses on agency rather than patiency
Akechi et al. (2018)	This study compared how 33 autistic and 45 non-autistic participants ascribed agency and experience to a variety of different types of entity. For both groups, robots were ascribed moderate agency (greater than dogs or human infants) but very low experience (similar to rocks). Attributions of “[m]oral blame positively correlated with agency, whereas moral consideration positively correlated with experience”
Al-Fedaghi (2007)	Al-Fedaghi takes Floridi’s Information Ethics further “by conferring moral value on personal information itself” and “moral consideration to the well-being of any personal information based on the moral concern for the welfare of its proprietor”
Allen and Widdison (1996)	Allen and Widdison consider computer contracts and legal personality from the perspective of protecting the computer’s users, including for convenience reasons. They encourage making some computer-generated agreements enforceable for the sake of “commercial pragmatism.” The legal precedent of personhood for other entities is considered, with autonomy being the relevant criterion; they see legal personality as “legally appropriate” at “a point” in the future
Anderson (2012)	Referring to “a family of theories we will refer to as ‘functional intentionality,’” Anderson argues that a machine “must first be shown to possess a particular moral status before it is a candidate for having genuine intentionality”
Andreotta (2020)	Andreotta argues that consciousness is a more important criterion for grounding “AI rights” than “superintelligence” or empathy. Andreotta argues that, “AIs can and should have rights—but only if they have the capacity for consciousness.” The “Hard Problem” of consciousness is seen as a key epistemic problem impeding “the AI rights research program”
Armstrong et al. (2012)	Armstrong, Sandberg, and Bostrom look an ’Oracle AI” approach to solving various AI issues. Discussing mind crime, they note that, “[i]t would be ideal if there were a specific level of detail beyond which the simulation would be conscious, and before which it is not,” but that achieving this would be difficult and the Oracle AI’s “answers may be inaccurate”
Arnold and Gough (2017)	Arnold and Gough depictions of personhood in several different films, with a focus on AI and robots
Asaro (2001)	This is a review of a book by Hans Moravec, “a leading researcher in robotics,” about a future where robots control the earth; Asaro describes it as “neither science nor fiction.” The book is criticized as giving only cursory and unconvincing discussion of many topics, including the moral consideration of artificial entities. Moravec apparently “argues that we should keep the robots enslaved… yet also makes the point that robots will be just as conscious and sensitive as humans”
Asekhauno and Osemwegie (2019)	This article “argues that each level of being,” including animals, plants, and AI “possesses some rights associated with it. It argues further that either all beings have rights, or they don’t.” It argues that accepting that these beings possess rights “poses the most existential and ontological threat to humanity and all of nature”
Ashrafian (2015a)	Ashrafian addresses the question of, “if robots do have rights, how should they interact with each other?” Various rights are proposed and compared to the universal declaration of human rights. Ashrafian argues for “a universal law of rights to recognise inherent dignity and the inalienable rights of artificial intelligences.” The suggestions are only intended to apply to the interaction between sentient robots and AI
Ashrafian (2015b)	Ashrafian focuses on outlining some philosophical considerations for determining robot responsibilities. Subsequently, Ashrafian briefly considers Roman law precedent to consider the rights that robots might have, noting that, as with Roman rights for slaves, the rights of robots might gradually expand over time, for practical reasons
Barfield (2015)	In this chapter, Barfield explores “whether the appearance of cyborgs and artificially intelligent machines will lead to discrimination from humans and if so, what laws exist to provide protection.” Barfield argues that artificial entities could be given serious consideration for legal personhood within “a few decades”
Barfield (2018)	“This paper reviews product liability and negligence tort law which may be used to allocate liability for robots that damage property or cause injury.” Different approaches to allocating liability are evaluated. The author concludes that, “[r]ights for robots may happen eventuality, but for now, determining liability for autonomous robots remains difficult”
Bartneck and Keijsers (2020)	After viewing videos of violence and abuse towards either a human or a robot, there was no significant difference between participant’s ratings of “the moral acceptability of the action, the violence depicted, the intention to harm, and how abusive the action was” but “[h]umans fighting back were seen as less immoral compared with robots fighting back”
Basl (2013a)	Basl argues that, “engaging in artificial consciousness research… might be unethical on grounds that it wrongs or will very likely wrong the subjects of such research.” The focus is on machines that are not “very much like us” or integrated into society. Interests and consciousness are seen as relevant criteria for patiency; Basl sees these as possible. Basl argues from both a deontological and consequentialist perspective
Basl (2013b)	This article makes similar arguments to Basl’s (2014) article. Several short thought experiments are used to critique alternative views, such as the use of religious views for determining moral patiency
Basl (2014)	The author argues that “current machines” do not possess the interests that would make them “moral patients,” even if you assume that, “consciousness is not always a necessary condition for having welfare.” However, “[o]nce artificial consciousnesses exist,” they will have interests that make them moral patients
Beckers (2018)	Beckers argues that, “even if we have good reason to believe that it is very unlikely, the mere possibility of humanity causing extreme suffering to an AI is important enough to warrant serious consideration.” Bekker notes that there is a technological assumption (“that an AI could become superintelligent”) and an ethical assumption (“that an AI could suffer”). Bekker argues that, “the possibility for an AI to experience supersuffering takes precedence over the expected benefits that an AI will produce for mankind” and that, “[h]umanity should not attempt to create a conscious AI”
Belk (2018)	Belk explores “who and what may be owned and by whom or what.” Belk argues that, with “consciousness, self-awareness, sentience, and sapience… It is quite imaginable that human-appearing and human-acting robots will be granted similar or greater protections than those now extended to animals”
Bennett and Daly (2020)	Bennett and Daly consider whether robots and AI “should be considered as legal persons.” They “analyse the regulatory issues raised by robot rights through three questions: (i) could robots be granted rights? (ii) will robots be granted rights? and (iii) should robots be granted rights?” Previous contributions are summarized; the authors do not argue for particular answers to these questions
Beno (2019)	Beno includes “a literature review on the extent and nature of robots and robot rights.” They then conducted a survey of 647 Slovak citizens, which found “a higher acceptance of the use of robots in everyday life… and in households than in the work environment,“ though no questions were asked directly about robot rights.’”
Bess (2018)	Bess proposes “three moral categories” of future nonhumans – “animals,” “persons,” and “presumed persons” – the latter two of which could include some (at least partly) artificial entities. Bess emphasizes “dignity” and “equality among all persons,” so argues that full personhood is not necessary for rights. Bess also argues that sentient artificial entities could possess the same rights as humans
Bigman et al. (2019)	The authors summarize moral psychology research on human perceptions of various robot capabilities, mostly relating to robot agency, with a brief discussion of robot rights. They note that “mind perception” predicts moral judgments and that people already perceive machines as having some relevant capabilities
Biondi (2019)	Assuming that we have no moral obligations to intelligent technologies now but will do in the future, Biondi asks whether “we have current actual obligations to technologies that do not currently exist,” referring to this as the “Non-Identical Machines Problem,” building on Parfit’s non-identity problem
Birhane and van Dijk (2020)	The authors take a “post-Cartesian, phenomenological view in which being human means having a lived embodied experience, which itself is embedded in social practices.” They “deny that robots, as artifacts emerging out of and mediating human being, are the kinds of things that could be granted rights in the first place.” They see this debate as “focused on first world problems, at the expense of urgent ethical concerns”
Birmingham (2008)	This article discusses a number of ethical issues relating to AI. Birmingham argues that, “no matter how sophisticated, a simulation does not become the thing.” Hence, artificial entities “will forever remain machines, and thus we are free to do with these machines what we do with other machines: turn them on or off, create or destroy, modify or dismantle in any way that we will”
Bolonkin (2012)	This short chapter in a book on various futures topics summarizes several ethical issues relating to AI and robots and questions the idea that personhood and intelligence are limited to humans. The subject is also mentioned briefly in another chapter in the same book: “The Natural Purpose of Humankind Is to Become God”
Bostrom (2014)	Though several sections of the book have some relevance to the wellbeing or suffering of artificial sentience, the discussion of mind crime comes from a single chapter. The argument is that, “a machine superintelligence could create internal processes that have moral status,” such as “a very detailed simulation.” They could be “subjected to various stimuli” or destroyed. Bostrom notes that, “the number of victims… might be orders of magnitude larger than in any genocide in history”
Bostrom et al. (2016)	The authors consider the “normative implications for governance and global policy” of superintelligence. The discussion of mind crime is similar to that in Bostrom’s (2014) book. Given the vast number of potential artificial sentient minds, the authors note that, “[t]he welfare of digital minds, therefore, may be a principal desideratum in selecting an AI development path”
Brey and Søraker (2009)	This chapter summarizes past discussions on a wide range of topics. There are a few paragraphs focused on the moral consideration of artificial entities, such as summarizing Floridi’s theory of Information Ethics
Briggs (2015)	Briggs summarizes a set of experiments which found that, “humanoid or non-humanoid appearance” did not “significantly affect reactions and/or agency ratings given toward the robot.” Briggs hypothesizes that, “the observed behavior of a robotic agent may be a more powerful determinant of the degree of agency and patiency people ascribe to robotic agents, rather than mere appearance”
Briggs et al. (2014)	Briggs et al. conducted an experiment which found evidence that, “humanoid appearance does not significantly affect the behavior of human operators in the task. Agency ratings given to the robots were also not significantly affected.” They hypothesize that, “actions speak louder than looks” in judgements of robots
Broman and Finckenberg-Broman (2018)	Broman and Finckenberg-Broman discuss several issues related to “Robotics/AI Legal Entit[ies].” They argue that current legal precedents could apply to artificial entities, if they are “competent to make necessary decision(s),” i.e. autonomous. Rights and obligations are seen as interrelated. Some current robots are seen as meeting these criteria, so “legal personality” is encouraged
Bryson (2012)	Bryson’s argument and focus seems to be the same as in their (2018) paper with a similar name
Bryson (2018)	Bryson argues that, “societies constantly reconstruct… ethical systems. Consequently, the place of AI systems in society is a matter of normative, not descriptive ethics.” Bryson argues that, “while constructing AI systems as either moral agents or patients is possible, neither is desirable… We are therefore obliged not to build AI we are obliged to”
Bryson et al. (2017)	The authors “review the utility and history of legal fictions of personhood” and “conclude that difficulties in holding ‘electronic persons’ accountable when they violate the rights of others outweigh the highly precarious moral interests that AI legal personhood might protect.” The authors comment that artificial entities may never become conscious and that creating conscious AI systems is undesirable
Calo (2016)	Nine legal case studies are used to explore “the role of robots as the objects of American law” and “robots as the subjects of judicial imagination”
Calverley (2011)	The article briefly considers “natural law” and “legal positivism” (developed by Bentham) as two contrasting theories of law as the context for the consideration of granting legal rights to machines. Calverley argues that if a machine has “autonomy,” then “we could assert the machine is the equivalent of a human in terms of its being held responsible,” regardless of whether or not it was “phenomenally conscious”
Cappuccio et al. (2020)	This paper uses virtue ethics and “social recognition theory” to argue that, “social robots should be credited as moral patients… because this is what a humane and compassionate agent would habitually do in their social interactions and because the opposite behavior would not be compatible with a virtuous lifestyle and moral flourishing”
Cave et al. (2019)	Cave et al. argue that creating intelligent machine moral agents “may lead to our granting them status as moral patients,” which, “runs the risk of creating new moral duties for humans, duties that may constrain us in important ways and expand our own moral responsibilities.” “Self-awareness” is presented as a more likely reason for granting them moral consideration than consciousness; they argue that some artificial entities may already meet this criterion
Celotto (2019)	This law journal article argues that the autonomous decision-making of AI has recently expanded, so “we are really at the point of having to write” laws about robot rights; they are “profoundly changing the rights of human beings” and “starting to formulate their own machine rights”
Čerka et al. (2017)	The authors explore “whether Systems of Artificial Intelligence (SAI)” can be granted legal personality.“ Several different legal and philosophical approaches and possible pathways to rights are evaluated. They criticize approaches focusing on the “metaphysical nature of the entity” as being narrow, though argue that the “nature” of the entity should not be neglected
Chernyak and Gary (2016)	Chernyak and Gary “asked [80] 5- and 7-year-old children to interact with a robot dog that was described either as moving autonomously or as remote controlled.” The autonomous robot “caused children to ascribe higher emotional and physical sentience to the robot, to reference the robot as having desires and physiological states, and to reference moral concerns as applying to the robot”
Chesterman (2020)	Evaluating the growing literature on the possibility that AI systems will gain sufficient autonomy and capabilities that they could be granted personhood, Chesterman argues that, “although most legal systems could create a novel category of legal persons, such arguments are insufficient to show that they should”
Chinen (2016)	Chinen focuses on legal responsibility for machines, as autonomous moral agents. Chinen briefly discusses personhood, summarizing several past legal and philosophical contributions
Chomanski (2019)	This article argues that, “it is unethical to create artificial agents possessing human-level intelligence that are programmed to be human beings’ obedient servants.” This is because “creators cannot help but evince an objectionable attitude akin to the Aristotelian vice of manipulativeness,” rather than because of the consequences that might arise, which could be positive or negative
Chopra (2010)	Chopra dismisses “civil rights for robots” as “fanciful” and “the stuff of good, bad, and simplistic science fiction” but nevertheless argues for the granting of “the status of a legal agent” to computer programs to protect “those that employ and interact with them”
Church (2019)	This book chapter discusses a range of interrelated topics, arguing, for example, that with regards to robots, “we should be less concerned about us-versus-them and more concerned about the rights of all sentients” and “harnessing this diversity to minimize global existential risks.” Comparison is made to previous rights expansions and “a human shield or figurehead monarch/CEO” is suggested as a method to obtaining rights for new “mind-types”
Coeckelbergh (2010a)	This article argues that appearances, rather than “proof of mental properties,” are used to judge whether robots should be seen as moral agents and that this is acceptable. Though the focus is on agency, Coeckelbergh notes briefly that the argument also applies to patiency
Coeckelbergh (2010b)	Coeckelbergh proposes a “social-relational” approach to granting moral consideration to artificial beings and critiques ontological approaches in consequentialist, deontological, and virtue ethicist thinking. Moral consideration is extrinsically “attributed to entities within social relations,” though the entity’s features are still used “as criteria on which we base our moral consideration”
Coeckelbergh (2013)	This review of Gunkel’s (2012) book identifies a number of contradictions. Firstly, Gunkel rejects Descartes but adopts a Cartesian approach. Secondly, “Gunkel wants to avoid ‘anthropocentric’ domination” but “presupposes that moral consideration is something that is and should be under human control.” Thirdly, Gunkel’s argument that “machines have a face” is not explained
Coeckelbergh (2014)	Coeckelbergh develops their “non-Cartesian,” “relational” approach to the question of whether we should “give moral standing to machines,” critiquing the traditional reliance on the “properties” of machines. Coeckelbergh critiques Gunkel’s approach that relies on Levinas’ concept of the “face.” Coeckelbergh believes that, “[r]obots are already part of our form of life, and this should be taken into account,” even though we might object in principle to granting robots moral consideration
Coeckelbergh (2018)	Some empirical psychology research is reviewed, followed by ethical discussion of “the ethics of empathizing with robots” and “the moral standing of robots.” Coeckelbergh “recommends first trying to understand the issue by means of philosophical and artistic work that shows how ethics is always relational and historical, and that highlights the importance of language and appearance in moral reasoning and moral psychology”
Coeckelbergh (2020)	As elsewhere, in the chapter on “Just Machines?” Coeckelbergh develops a social-relational approach to the question of whether we give AI moral consideration, critiquing the traditional reliance on the “properties” of machines. Patiency is only considered briefly, alongside agency. This is a small part of a book on a number of topics related to “AI Ethics”
Craig et al. (2019)	The authors’ experiment compared “warning compliance-gaining” and “obligation compliance-gaining” strategies for considering robot rights. The latter had more favorable ratings for “perceived caring” and “task/social attraction”
Dall’Agnol, Darlei (2020)	Dall’Agnol argues that “there are basic, intrinsic rights to personhood, which… allow us… to attribute rights to artificial agents.” Dall’Agnol cites science fiction and poses rhetorical questions. Robots are considered among other nonhuman or partly human entities that could plausibly be granted rights. Agency and “capacity for action” are presented as relevant criteria for determining whether entities warrant rights
Damholdt et al. (2020)	Damholdt et al. develop “the attitudes towards social robots scale (ASOR)” with 25 questions by surveying 339 people, conducting factor analysis, and carrying out 10 interviews. ASOR comprises “three distinct facets of ascription of capacities to social robots”; “ascription of mental capacities,” “ascription of socio-practical capacities,” and “ascription of socio-moral status”
Danaher (2020)	Asking whether robots can have “significant moral status,” Danaher presents a theory of “ethical behaviourism,” “which holds that robots can have significant moral status if they are roughly performatively equivalent to other entities that have significant moral status.” This is presented as “an essential feature of day-to-day ethical practice.” The theory “is then defended from seven objections,” including Gunkel’s
Darling (2016)	Darling “explores the near-future possibility” of regulating behavior towards robots. Darling summarizes numerous empirical studies on the interaction of humans with artificial entities, arguing that there is a strong tendency towards anthropomorphism. Darling argues that animal rights laws follow “popular sentiment rather than consistent biological criteria” and robot rights laws could do the same
Davidson et al. (2019)	This is a pre-registration of a proposed psychological study. The authors summarize some previous empirical research that “indicates that children will protest a robot being treated unfairly by a human”
Davies (2011)	Davies analyses issues concerning “the ownership of computer generated works within patents and copyright” and concludes that “the current regime is woefully inadequate.” Davies expresses “little doubt” that computers that write other “advanced AI programs” are the entities “best entitled” to the relevant property rights. Though relevant issues are presented as likely to arise in the future, proactive legal changes are encouraged
Dawes (2020)	Dawes considers various possible “speculative rights” issues if artificial general intelligence (AGI) is developed. Dawes presents it as unclear whether AI will ever be conscious, given uncertainties in current consciousness research. Some relevant discussion and news events relating to AI rights are summarized and Dawes predicts that, “[t]he most likely future development (assuming a successful control scenario) is neither the extreme of full inclusion nor full exclusion”
De Graaf and Malle (2019)	Analyzing participants’ descriptions of robots’ behavior, De Graaf and Malle found that, “people use the same conceptual toolbox of behavior explanations for both human and robot agents, robustly indicating inferences of intentionality and mind.” However, participants were more likely to explain humans’ behavior with reference to their mental states and more likely to explain robots’ behaviors with reference to background facts, such as about the robots’ programming
DiPaolo (2019)	DiPaolo discusses personhood and rights for robots, with reference to science fiction, especially the film Westworld. DiPaolo advocates for modification of legal frameworks, including “responsibilities” for the owners of androids, and argues that humans should not abuse androids. The reasoning is not clearly stated
Dixon (2015)	Dixon argues that, “issuing rights to AI would actually be more for the benefit of humans than of robots” due to human empathy for mistreated robots. These concepts are discussed with reference to science fiction films. Dixon assumes that robots cannot have empathy
Dracopoulou (2003)	This article discusses in some detail the concept of “the value of life,” arguing that, all “persons,” not depending on species, have very special, intrinsic value.“ It argues that ”conscious robots” could have equivalent “moral status” to humans. It also discusses whether it would be morally acceptable to create conscious robots
Drozdek (1994)	Drozdek argues against personhood for computers by noting that this might lead to negative consequences (including practical difficulties like updating their software) and by suggesting that the criteria for personhood (consciousness, interests, autonomy and so on) could never be proven to be present in a computer. Kant, Plato, and others are invoked to argue that “moral dimension in man occupies a pre-eminent position”
Drozdek (2017)	Drozdek “addresses the problem of possible rights for superintelligent systems by using a distinction between moral dimension and rational dimension in human beings and proposing to endow artificial systems only with rational dimension.” Drozdek presents the need for moral consideration of artificial entities as a potentially problematic by-product of designing intelligent tools
Erhardt and Mona (2016)	Although considering legal stats, Erhadt and Mona focus on philosophical issues and consciousness research. Erhadt and Mona see some of the criteria as already partially fulfilled and conclude that, “although no existing artificial intelligences are considered legal entities, but this is likely to change in the next few decades”
Estrada (2018)	Estrada considers how Alan Turing’s principle of “fair play for machines” can integrate the debate over robot rights within the AI alignment literature. Estrada focuses more on “legal agency” than on sentience and argues that, “extending rights to service robots operating in public spaces is ‘fair’ in precisely the sense that it encourages an alignment of interests between humans and machines”
Estrada (2020)	Estrada critiques “human supremacism” in AI ethics, especially the works of Joanna Bryson and colleagues, drawing on “feminist, postcolonial, and critical race theory,” “animal rights and environmental ethics,” and various other theories. Gunkel, Darling, and environmental ethics precedent are cited for evidence that “biological factors should not be treated as prima facie justification for the exclusion of artificial agents from the moral community”
Fagan (2019)	Fagan argues that “AI rights recognition will occur, if at all, as a result of consensus-building among the economic beneficiaries of AI rights creation” but that, “Inasmuch as existing law can efficiently balance the costs of misalignments with the benefits of innovation and AI proliferation, then AI rights should not be granted despite calls from special interest groups”
Floridi (1999)	This paper outlines Floridi’s theory of Information Ethics. Consequentialism, Contractualism and “Deontologism” are critiqued as “unable to accommodate” problems in computer ethics. Information Ethics is presented as “the philosophical foundation” of computer ethics. Information Ethics “evaluates the duty of any rational being in terms of contribution to the growth of the infosphere,” including non-sentient artificial entities. Information entropy constitutes “an instance of evil”
Floridi (2002)	This paper expands on Floridi’s theory of Information Ethics, developing “the thesis that the minimal condition of possibility of an entity’s least intrinsic value is to be identified with its ontological status as an information object. All entities, even when interpreted as only clusters of information, still have a minimal moral worth qua information objects and so may deserve to be respected”
Floridi (2005)	This paper summarizes various components of Information Ethics and addresses several criticisms. The question “What counts as a moral patient, according to IE?” is answered with: “All entities, qua informational objects, have an intrinsic moral value, although possibly quite minimal and overridable, and hence they can count as moral patients, subject to some equally minimal degree of moral respect understood as a disinterested, appreciative and careful attention”
Fox (2018)	Asking whether sexbots can be abused, Fox considers various methods of understanding moral status. Kant’s focus on autonomy and rationality is presented as the conventional account and is critiqued, e.g. noting that non-Western cultures value other criteria more. “Harm” and interests are presented as more important. Fox sees the social-relational account as most promising, though argues that it needs further exploration
Frank and Nyholm (2017)	Frank and Nyholm explore “whether it is conceivable, possible, and desirable that humanoid robots should be designed such that they are capable of consenting to sex.” They argue affirmatively to all three questions, both for the benefit of robots’ own wellbeing (if they become “sophisticated enough to enjoy a certain degree of consciousness”) and wider societal implications
Fraune et al. (2017)	This study “puts participants into two competing teams, each consisting of two humans and two robots, to examine how people behave toward others depending on Group (ingroup, outgroup) and Agent (human, robot) variables.” The ingroup and humans were favored, but the effect of group was stronger; “participants preferred ingroup robots to outgroup humans”
Freier (2008)	In this study, children interacted with an animated human from the videogame Half-Life 2. Half the participants saw the agent react negatively to a verbal insult by a researcher. “47% of the children in the control [no response to insult] condition judged the verbal insult as a moral violation,” compared to 90% in the of the children in the reaction condition. Most children referred to the animated agent in their evaluations
Friedman (2019)	This article takes a “human perspective” and “relational account” to examine the moral consideration of social robots, arguing that “what matters is not whether robots are actually phenomenally conscious… but whether we view them as possessing the property of phenomenal consciousness.” It argues for “granting negative rights to robots” to protect “the moral fibre and quality of human societies”
Galanter (2020)	Galanter uses their theory of “complexism,” alongside various theories from moral philosophy and research on consciousness, to consider moral consideration of artificial entities. Galanter concludes that, “there is nothing that presently proves machine sentience is impossible… As such, a sense of due diligence should oblige us to extend patiency to apparently aware machines as our own moral obligation”
Gamez et al. (2020)	The authors focus mostly on moral agency but also discuss of moral patiency. They argue that, for artificial entities “with whom we interact socially, insofar as their process of learning to behave ethically models the acquisition of virtue… from the perspective of virtue ethics this amounts to being a full moral agent, or close enough”
Gerdes (2015)	Gerdes examines “human technology relations through the lens of sci-fi movies.” Steven Spielberg’s film Artificial Intelligence apparently explores ideas comparable both to Gunkel’s social-relational perspective and Turkle’s personhood perspective on granting moral consideration to artificial entities
Gerdes (2016)	Gerdes “stick[s] to a human centered framework and introduce[s] a moral philosophical perspective, primarily based on Kant’s Tugendlehre and his conception of duties as well as the Formula of Humanity, which also holds a relational perspective.” Gerdes comments that “destruction” or “violent and cruel” treatment of animals, nature, and inanimate objects “is always only a duty of the human being to himself”
Gittinger (2019)	Gittinger discusses several ethical issues, focusing briefly on robot rights, with reference to science fiction. Consciousness and autonomy are presented as relevant criteria. Personhood is discussed, but mostly in terms of the ethical responsibilities of AI and robots themselves
Gloor (2016b)	This is the paper where Gloor outlined some of the scenarios summarized in Sotala and Gloor (2017). Hence, the basic argument – that artificial sentience could suffer in astronomical quantities and that this would be an extremely bad outcome – is similar. Additional cited risks for suffering among artificial sentience include intentional punishment, “programming mistakes, unanticipated loopholes or side-effects,” and “black swans”
Gordon (2020)	Gordon argues that, “[w]hether intelligent robots are, in fact, moral beings is not a matter of popular vote or social opinion. Rather, their moral status depends on empirical factors.” Adopting in part a “neo-Kantian” approach, Gordon presents and defends several arguments for why intelligent robots “should be entitled to moral rights once they are capable of moral reasoning and decision-making”
Gray and Wegner (2012)	The authors found in a study of 130 students and members of the public in Cambridge, MA that participants attributed similar agency to “humanlike” and “mechanical” robots, but attributed greater experience (capacities to feel pain and fear) to the humanlike robots. This study and a follow-up study also found evidence that artificial entities with higher levels of experience are more unsettling to humans
Gregory (2012)	Gregory considers whether “the actions the player takes within the game entirely on their own merits.” From consequentialist and deontological perspectives, Gregory concludes that, “there is little ability to justify classifying video-game entities as entities deserving of moral standing” but that, from the perspective of virtue ethics, “a player is able to use the game as an opportunity to practice their own sense of morality”
Gualeni (2020)	Gualeni explores whether artificial entities in “virtual environments” like “digital games and training simulations” warrant moral consideration, arguing that having “interests” is more important than sentience per se; this requires “autonomy and integrity.” Gualeni concludes that, “[i]t is not morally viable to use autonomous artificial intelligences in environments where, by design, their autonomy will largely be suppressed”
Gunkel (2007)	This is an early paper by Gunkel outlining similar arguments to those expounded more fully in his books, such as the need to “think otherwise” about moral agency and patiency. Gunkel argues that machines have been neglected in discussions about moral consideration
Gunkel (2012)	The book critiques René Descartes, who “came to associate the animal with the machine.” The first chapter evaluates the moral agency of machines, the second chapter evaluates their moral patiency, and the third chapter uses Emmanuel Levinas and Jacques Derrida to “think otherwise” and deconstruct the “agent–patient conceptual opposition.” No clear answer is offered to “The Machine Question”
Gunkel (2013)	This is a book review of Mark Coeckelbergh, Growing Moral Relations: Critique of Moral Status Ascription (Basingstoke, UK: Palgrave Macmillan, 2012). Rather than relying on ontological criteria, “Coeckelbergh’s alternative approach to moral status ascription can be described by three terms: relational, phenomenological, and transcendental”
Gunkel (2014)	Here, Gunkel advances similar arguments to elsewhere (e.g. 2012, 2018, critiquing existing approaches and using Emmanuel Levinas as precedent for “questioning the very protocols of inclusion/exclusion that have organized and structured moral philosophy.” Despite the framing of the article, Gunkel again critiques existing perspectives without making a strong case that machines should be considered morally
Gunkel (2015)	This chapter makes similar arguments to Gunkel’s other work, but applied specifically to robot care-givers
Gunkel (2018a)	Gunkel surveys previous contributions to the debate and critiques all four possible combinations of views on the two questions of (1) whether robots can have rights and (2) whether robots should have rights. The final chapter uses Emmanuel Levinas’ ideas to “think otherwise” about the question, critiquing the terminology and ontological criteria (e.g. sentience, consciousness, autonomy) used by previous contributors
Gunkel (2018c)	Gunkel advances very similar arguments to their 2018 book and uses a similar structure
Gunkel (2018d)	In this chapter, Gunkel uses a similar method of analysis to elsewhere, asking “Should machines have rights?” “not on the basis of what things are but on how we decide to relate and respond to them”
Gunkel (2018b)	As elsewhere, Gunkel critiques existing approaches to answering “The Machine Question” and advocates for “thinking otherwise.” Gunkel’s “objective in this chapter has not been to resolve the question of machine moral standing once and for all, but to ask about and evaluate the means by which we have situated and pursued this inquiry”
Gunkel (2019a)	As with Gunkel’s other papers, Gunkel critiques the reliance on ontological criteria for the attribution of “moral and/or legal status of others” and encourages “thinking otherwise,” using empirical evidence of “extrinsic social relationships” instead
Gunkel (2019b)	Gunkel advances similar arguments to Gunkel’s arguments elsewhere but focuses on the specific issue of military robots and argues that military animals and military robots “are not considered purely property in practice”
Gunkel (2020a)	This article outlines similar arguments to those outlined by Gunkel elsewhere but responds specifically to an article offering “A Confucian ethical perspective” and noting that there are many similarities between that article and Gunkel and Coeckelbergh’s social-relational approach
Gunkel (2020b)	This chapter follows a similar structure and argument to Gunkel’s other works, critiquing “current thinking (or lack of thinking)” about robot rights, mapping “the terrain of the set of available answers that have been provided in the existing literature,” and offering “an alternative way” of engaging with the issues
Gunkel and Cripe (2014)	As with Gunkel’s other papers, Gunkel and Cripe advocate for “social-relationalism.” They conclude that whether AIs should be seen as “moral subjects” “cannot be answered definitively and finally with a simple ‘yes’ or ‘no.’ The question will need to be asked and responded to repeatedly in specific circumstances and the answers that come to be provided will necessarily be provisory and open to ongoing debate”
Hagendorff (2020)	Hagendorff addresses some conceptual issues in animal rights and robot ethics. Hagendorff is concerned that, “robot ethics perpetuates the… isolation and definition of a particular entity possessing a moral status,” which “excludes all those entities which… do not fit exactly to the pre-specified definition”
Hale (2009)	Hale argues that the moral considerability of “technological artefacts” is tied directly to their value to humans, such as being an art object or a resource, “and not to assertions of the wills of their creators.” Hale argues that “everything in the world is morally considerable” but that to consider technological artefacts “qua technological artefacts,” for their technical aspects, “is to double-count”
Hall (2005)	In this study, “a synthetic character that exhibits the behaviour characteristic of feeling pain was constructed” and the responses of eight users were examined. All the users inflicting pain on the character at least once, but users seemed less willing to inflict pain in a group setting than in an individual setting
Hallqvist (2018)	Hallqvist analyzes the “borders between humans and hubots (humanoid robots) in the Swedish television series Äkta människor (RealHumans)” and discusses its implications for robot rights
Hanák (2019)	This article summarizes the works of previous scholars and adds short “I agree” or “I disagree” statements as commentary, focusing on “laws that currently apply in the Czech Republic.” The author encourages liability for AI going to “its creators,” rather than the AI itself. The ability to suffer is seen as an important, relevant criterion
Hartmann (2017)	Hartmann summarizes some studies that provide evidence that people tend to perceive video game characters and other artificial entities as alive, having mind, and deserving some moral consideration. This is used as part of an argument refuting “the view that users enjoy virtual violence primarily because they are constantly aware that ‘this game is not real.’”
Himma (2003)	Himma discusses several claims that have been made in computer ethics, some of which relate to the moral patiency of computers and other artificial entities. Himma argues that computers do not currently have moral standing, though any future “instantiation of consciousness and understanding” in artificial entities would give them moral standing
Himma (2004)	Himma critiques several components and assumptions Floridi’s Information Ethics, such as that the historical process of moral circle expansion justified further expansion and that Floridi’s “information objects” are “radically different” from “the entities they describe”
Hoffmann and Hahn (2020)	Hoffman and Hahn argue that defining suffering is difficult, as is “knowing if someone or something that appears to be suffering is indeed actually doing so.” They argue in favor of “[t]hinking otherwise” and “an extreme and carefully articulated version of relativism.” They consider policy implications, such as that, “once AI can participate in the moral discourse, arguing credibly and convincingly for its rights, they shall be granted”
Hogan (2017)	This article evaluates the arguments put forward in Gunkel (2012) and argues that, “the machine question and the animal question are different questions,” including by arguing that these questions do not “phenomenologically appear as the same question in particular situations.” Hogan argues that the ideas of “aliveness” and the distinction between moral agency and patiency are “foundational to our very idea of human ethics”
Holder et al. (2016)	The authors discuss several legal issues related to AI and robots, including “Robot-generated rights.” They conclude that, “rights relating to the types of IP that are protectable do not need to be updated as yet,” but that society may need to engage with the question of robot rights in the future
Holm and Powell (2013)	This article explores several issues in “synthetic biology,” which is described as having a “tendency to blur boundaries between supposedly discrete ontological categories, such as between organism and machine, living thing and artifact, ‘the natural’ and ‘the artificial’.” Holm and Powell summarize some relevant discussions by other contributors as to whether “the products of synthetic biology” have interests and warrant moral consideration
Holy-Luczaj and Blok (2019)	Holy-Luczaj and Blok consider whether “hybrids,” “entities crossing the ontological binarism of naturalness and artificiality,” have moral considerability. Drawing on environmental ethics, they argue that, “it is primarily the ability to serve other beings by performing certain of their functions that belong to their identity, which should qualify a being to have moral considerability.” This “challenges the strong opposition between the ethical status of natural beings and that of artifacts”
Hu (2018)	Several legal precedents for “robot criminal liability” are discussed; “robots as products” (with liability falling on the human users), which will be challenged as tech develops, “robots as animals” (which could result in some legal protections, if not liability), and “robots as corporations,” and “robots as humans.” Hu argues for legal liability, but no opinion is offered on rights
Hughes (2005)	This representative survey of members of the World Transhumanist Association found that, “70% support human rights for ‘robots who think and feel like human beings, and aren’t a threat to human beings.’”
Huttunen et al. (2010)	The authors focus mostly on legal liability for robots, though this is explicitly tied to the idea of rights. Intelligence and autonomy are presented as the key criteria for granting this. The precedent of slavery is considered. The authors propose an “insurance framework,” whereby “a machine can become an ultimate machine by emancipating itself from its manufacturer/owner/operator”
Inayatullah (2001)	Inayatullah highlights how different philosophical traditions from across the globe might be more or less receptive to robot rights. It also highlights some uncertainties regarding legal precedent for robot rights. Many of the arguments are similar to McNally and Inayatullah (1988)
Inyashkin (2016)	Inyashkin “explores several works by Isaac Asimov” and their discussion of human and robot rights. The “robots described in the fiction have a lot in common with slaves, while they might potentially become equal members of a human society”
Jack et al. (2013)	Jack, Dawson, and Norr conducted two neuroimaging studies testing humanizing and dehumanizing effects, through comparisons between humans, animals, and machines. Both studies found that, “human and humanizing conditions were associated with relatively high activity in the DMN [default mode network] and relatively low activity in the TPN [task positive network]”
Jackson Jr. (2018a)	Jackson asks whether “a human-level AI can terminate its simulations of other minds without committing ‘mind-crimes’” and several related ethical issues. Jackson suggests that, “purely symbolic artificial consciousness could be equivalent to human consciousness.” However, the paper is presented as a “caveat” to Jackson’s previous (2018b) paper arguing that “there need not be an ethical problem in switching off a purely symbolic artificial consciousness”
Jackson Jr. (2018b)	Jackson argues that, “[a]rtificial consciousness need not be equivalent to human consciousness, and there need not be an ethical problem in switching off a purely symbolic artificial consciousness,” for example if “artificial consciousness does not have any subjective experiences approaching human consciousness”
Jackson and Williams (2019)	Focusing research relating to “perceived social agency and perceived moral agency,” Jackson and Williams summarize some research of relevance to the moral consideration of artificial entities. For example, they summarize one study where they “saw significant decreases in the robot’s likeability” when it refused a “less morally objectionable” human command in a “less polite” manner. They argue that the “phrasing” used by robots “can be just as important as the message itself”
Jaynes (2020)	This article analyzes the rights that could be granted to “non-biological intelligence possessing legal and civic duties akin to those possessed by humanity today,” presenting relevant jurisprudential evidence. The article encourages “capabilities-based altruism” towards artificial entities and the urgent granting of relevant rights
Johnson and Miller (2008)	This paper focuses mostly on moral agency, but briefly cites’ Sullins’ (2005) comments about moral consideration to critique “determinist” thinking about artificial entities, arguing that artificial “functional equivalents” are not the moral equivalents of other entities
Johnson and Verdicchio (2018)	Johnson and Verdicchio critique the analogy between animals and robots from a number of perspectives, including the moral status of the entities themselves (emphasizing that “animals suffer and robots do not” and “even those of the future will not suffer”), their legal status, and the “Impact on social behaviour: how we treat robots and each other.” There is some review of the empirical evidence
Jowitt (2020)	Jowitt argues defends “a moral foundation for the recognition of legal personhood for AI, requiring the status to be granted” if they reach a threshold of “bare, noumenal agency in the Kantian sense” against counterarguments, such as by Bryson et al. (2017). Jowitt does not see this threshold as having been met yet. Jowitt then uses this framework to evaluate “proposals currently making their way through the legislatures of the UK and European Union”
Kaminska (2016)	Kaminska analyzes 20 films from the Guardian’s list of top 20 films about AI for their discussion of rights for robots, especially the rights to life, equality, and self-determination. Common themes are extracted. Though the paper does not present a moral argument, the author encourages that they be granted rights once their capabilities advance sufficiently
Kaufman (1994)	Criticizing a common view among environmental philosophers, Kaufman argues that, “either machines have interests (and hence moral standing) too or mentality is a necessary condition” for moral consideration. Additionally, Kaufman argues that “the aspect of mentality necessary for having interests is more complicated than mere sentience”
Kelley and Atreides (2020)	Kelley and Atreides describe “a laboratory process for the assessment and ethical treatment of Artificial General Intelligence systems that could be conscious and have subjective emotional experiences ‘theoretically.’” They claim that, “there are now systems—including ones in our lab—that… are potentially conscious entities” and note that, “[t]he fundamental assumption of this Protocol is that the treatment of sapient and sentient entities matters ethically”
Khoury (2016)	Different approaches to the rights of and liabilities for “human-like robots” are evaluated. Khoury believes that they are “not alive” and if they go “rouge,” would need to either be “fixed” or “terminated”
Kim and Petrina (2006)	Kim and Petrina discuss the computer game The Sims and place this in the context of some previous discussions of robot rights
Kiršienė and Amilevičius (2020)	The authors examine AI legal issues in the context of the European Parliament’s proposals, from a legal and technological perspective. Autonomy and embodiment are discussed as criteria for personhood. They argue that, “while there may be future conditions to justify or even necessitate AI personhood, doing so now appears to be technically premature and is likely to be inappropriate”
Klein (2016)	This paper explores questions of moral patiency and moral agency of robots from a utilitarian perspective. Klein argues that, “to believe that ‘moral’ equates to ‘natural’… would be a case of the naturalistic fallacy.” Whether an entity’s “preferences/sense of well-being“ are from natural causes or programming, “we ought to recognise them the same.“
Klein (2019)	Klein critiques human and “bio-exceptionalism,” where “one type of entity is considered exceptional by definition or a priori.” This exceptionalism includes Singer’s use of a specific understanding of consciousness; other contributors such as Bryson, Coeckelbergh, and Sparrow are also critiqued. Klein argues that “adherence to ontological properties as criteria for ethical consideration” is “the only workable” approach
Klein and Lin (2018)	Klein and Lin respond to Kathleen Richardson’s arguments in favor of a ban on sex robots. The focus is on the effects of sex robots on human women and children. There is a brief discussion of artificial sentience in the conclusion, noting that “whether it is a sentient human or a machine in any given situation, changes very little in terms of moral consideration”
Kljajić (2019)	This MA thesis summarizes literature on various topics related to the moral status of artificial entities. Anthropomorphism is presented as a risk and consciousness seen as a key criterion for granting moral consideration. Kljajić sees artificial consciousness as possible, but neither consequentialist nor deontological theories as entirely adequate for evaluating artificial entities’ moral status
Kolling et al. (2016)	Although not explicitly focusing on moral consideration of artificial entities, this article summarizes and evaluates research in adjacent issues, such as emotional attachment to robots and moral qualms about damaging them
Kovic (2020)	This paper explores the risks associated with space colonization, which “could result in enormous disvalue,” and how these risks could be mitigated. Some of the discussion focuses on the risks for artificial sentience, noting that ”colonizing space would make [related] adverse outcomes more probable”
Krämer (2020)	Though not exploring moral consideration of artificial entities per se, Krämer argues that, “robots cannot be rightly seen as possessors of dignity” and this is presented as affecting questions of “how humans should treat robots, and how robots should treat each other”
Krebs (2006)	This article “examines the influence of mangas and animes on the social perception and cultural understanding of robots in Japan.” Robot rights are discussed, as are relevant cultural factors in Japan
Kunnari (2020)	Kunnari “examines what kind of moral patiency and agency an android character Lore is granted in the science-fiction television show Star Trek: The Next Generation” and “employs philosophical theories of morality in analyzing Lore’s treatment by different characters”
Kuran (2020)	Kuran reviews “the literature on the moral status of AI, emphasizing that although a majority of philosophers agree that AI could plausibly have moral status based on capacities, there is disagreement about the specific degree to which such AI has moral status.” Kuran argues in favor of a “capacity-based approach that recognizes AI as moral patients or agents depending on the context”
Küster and Świderska (2016)	In this experiment, Küster and Świderska compared responses to a human or humanoid robot being apparently either presented flowers or tasered. They found “a significant difference in the pairwise comparison between benevolent vs. malevolent intention towards the robot” but that “when the malevolent action was being carried out against the human, it was evaluated as more morally wrong than when carried out against the robot”
Küster and Swiderska (2020)	Küster and Swiderska found that, contrary to their previous experiments, “manipulation of intentions” (benevolent vs. malevolent) failed to affect mind perception.“ However, they found that ”benevolent intentions reduced dehumanisation of the patients.” They also found that, “[b]enevolent and malevolent intentions were evaluated respectively as morally right or wrong, but their impact on the patient was diminished for the robotic avatar”
Küster et al. (2020)	Küster, Swiderska, and Gunkel examined responses to “four popular online videos of a leading robotics company (Boston Dynamics) and one more familiar vacuum cleaning robot (Roomba).” The results suggest that, “unexpectedly human-like abilities might provide more potent cues to mind perception than appearance, whereas appearance may attract more compassion and protection”
Laukyte (2017)	Laukyte argues that, if future artificial agents meet the conditions currently required for group agents such as corporations to be granted legal rights, they too should be granted rights. This requires them to be seen as rational and interactive
Laukyte (2019)	Laukyte draws on Deep Ecology and environmental ethics to argue that “artificially intelligent entities” are “an integral part of that environment” and warrant moral consideration, regardless of their sentience or other potential ontological criteria
Laukyte (2020)	Laukyte looks at legal issues relating to “robot as product” and “robot as entity,” the latter of which relates to rights. Laukyte summarizes contributions arguing for or against robot rights and notes that, “there also is a shift underway towards a more nuanced discussion focused on specific rights, such as… a right to legal advice”
Laulhe-Shaelou (2019)	Criteria for rights such as consciousness and autonomy are discussed. The authors conclude that, “robots and AI cannot be granted all the same rights as humans” but that robots could be “entitled to a different set of rights, if any, that corresponds to the level of their consciousness, autonomy and rationality.” These issues are discussed with reference to EU law, but most citations are blog or magazine articles. Specific rights are proposed
Lavi (2019)	Lavi cites consequentialist and deontological thinkers on animal rights but criticizes the use of language that anthropomorphizes non-humans and defends giving greater moral standing to humans than animals or robots
Lee et al. (2019)	In this experiment, manipulations of the described moral patiency of artificial entities significantly affected participants’ ratings of their agency, but the manipulations of described moral agency did not significantly affect perceptions of patiency. Participants also rated entities as lower in patiency when they were described as not able to feel. Various other interactive behaviors and evaluations of the entities were examined
Leenes and Lucivero (2014)	This article describes legal precedent for regulation of technologies, considered through four separate categories of regulation types
Lender (2016)	Lender argues that, “if AI have phenomenal consciousness, they will necessarily hold morally relevant interests that must be accounted for and weighed against those of other sentient species. Using a Utilitarian moral framework,” Lender argues that, “ceteris paribus, the interests of conscious artificial superintelligent systems, should be prioritized over those of humanity,” which could potentially lead to human extinction
Levy (2009)	Levy reviews previous discussion of the moral and legal precedent for robot rights. Levy concludes that robots “cannot feel pain and suffering” but should nevertheless “be endowed with rights and should be treated ethically,” partly because ”robots will be regarded by us in some similar ways to those in which we regard other humans”
Levy (2012)	As in their 2016 paper, Levy examines various ethical issues relating to sexbots, including brief discussion of the impacts on the sexbots themselves
Levy (2016)	This article focuses on “love and sex with robots,” but the issue of robot personhood is discussed. Levy argues that, “a robot does not necessarily lack personhood just because it is built differently from a human, nor is that difference alone sufficient reason to deny a robot the same rights and protections ascribed to humans.” The legal precedents of the rights of corporations and gay marriage are discussed
Lima et al. (2019)	Korean youths were surveyed on their views about whether robots should be granted rights and moral consideration in response to five specific fictional scenarios. The results were mixed. The authors interpret the results as showing that, “children are primarily supportive of robot rights in scenarios that contain human–robot interactions at their core” but not where robots are independent. However, this distinction was not directly tested
Lima et al. (2020)	The authors asked online survey participants about “11 possible rights that could be granted to autonomous electronic agents of the future.” Respondents were opposed to most of these rights but supported the “right against cruel treatment and punishment.” The researchers also found significant effects from providing additional information intended to promote support for robot rights
Loh (2019)	Loh discusses “three fields of robot ethics,” one of which is the consideration of moral patiency. Loh focuses on this issue within the theoretical framework of “responsibility networks,” which is described as a “relational concept.” Loh concludes that artificial entities “could be identified as object or even addressee of one or more responsibilities… in this manner it is possible to integrate robots as moral patients in responsibilities.” Autonomy seems to be important for this
Lopez-Mobilia (2011)	In this study, “children were asked whether or not different kinds of nonhuman entities (dogs, trees, robots, dolls) were capable of a range of psychological states (e.g., thinking, feeling)” then “asked to judge the morality of actions that led to a negative consequence for a nonhuman target.” The results showed no “relation between psychological attributions and moral judgments,” though this could be due to the study methodology
Lupetti et al. (2019)	After summarizing previous contributions relevant to the discussion of robot citizenship, the authors summarize their findings from interviews with roboticists on “a small series of current urban robot challenges.” They argue for the importance of a “socio-relational perspective” in the design of robots
MacDorman and Cowley (2006)	MacDorman and Cowley argue that the “ability to sustain long-term relationships” is “the most compelling” benchmark for personhood, since other benchmarks are “trivial, subjective, or based on assumptions about moral universals.” This argument is related to various ethical and legal debates. MacDorman and Cowley seem to see this as possible and note that, “the most humanlike of robots will be best equipped for reciprocal relationships with human beings”
Mackenzie (2014)	As in other articles, Mackenzie argues that “[a]s creators of sentient, self aware beings designed for utilitarian purposes” (i.e. sexbots of the future), humans “owe them a moral duty to entrench measures to promote their agency, fulfillment and flourishing.” Some other related ethical issues relating to sexbots are discussed
Mackenzie (2016)	Mackenzie argues that, “humans owe sentient, self-aware sexbots an ethical duty as creators of sentient beings to protect them from harm and suffering,” as in their other articles
Mackenzie (2018)	Mackenzie argues that “future humans will want… sex robots customized to possess sentience and self-awareness” and “explores theoretical, ethical, and pragmatic aspects of the tensions involved in creating sentient beings for utilitarian purposes.” As elsewhere, Mackenzie argues that, “humans as creators should owe them a duty to protect their interests and to minimize their suffering”
Mackenzie (2020a)	Mackenzie offers “tentative suggestions for ways forward drawing on Tibetan Buddhism and the Tantric tradition… in order to foster a post-Singularity future where all sentient beings, including super-compassionate superintelligent robots, sexbots, humans and nonhuman animals, can flourish”
Mackenzie (2020b)	As in other articles, Mackenzie argues that, “humans as creators owe a duty of care to sentient beings they create,” because of “vulnerabilities” that may be created in artificial creations. This could include legislative restrictions on customization of sexbots and regulations on behavior
Magnani (2005)	Magnani’s argument, as it relates to the moral consideration of artificial entities, seems to be the same as in their 2007 paper
Magnani (2007)	Magnani argues that the distinction between moral agents and moral patients is unhelpful; the concept of “moral mediators” is more appropriate. Drawing on Kantian ethics, Magnani argues that, “in recent times, non-human beings, objects, and structures like technological artifacts and machines have acquired new moral worth and intrinsic values”
Malle (2016)	A number of previous contributors to relevant ethical discussions are summarized. Malle comments that, “As robots acquire increasing moral competence, especially moral judgment and decision making, it may well be unethical to deny them all moral standing. Their rights may be limited, however”
Massaro (2018)	This chapter argues that, “[t]he First Amendment may protect free speech rights for strong Artificial Intelligence (AI),” but not necessarily all AI, and addresses criticisms of this view
Massaro and Norton (2015)	This article examines existing legal precedent for free speech and how this might affect artificial agents. It is argued that “speaker humanness no longer may be a logically essential part of the First Amendment calculus,” though the authors present this as counterintuitive. The “interests of human… listeners” are mostly prioritized in the article
Mazarian (2019)	Mazarian analyzes and critiques philosopher Eric Schwitzgebel’s defence of the possibility of equal rights of AIs and humans. One critique offered is that, “considering possible metaphysical worlds,” it does not seem possible that robots could have “psychological states” without having a human body
McLaughlin and Rose (2018)	McLaughlin and Rose argue that sentience affects moral status but that it is an “open question” whether sentient or intelligent robots “will ever be technologically possible.” Related ethical issues are explored. McLaughlin and Rose’s study presented participants with a story about biological entities and behaviorally identical robots. Participants gave lower scores for whether the robots could feel “pain, itches, warmth, or pressure”
McNally and Inayatullah (1988)	McNally and Inayatullah explore some legal precedent for robot rights and considers how and why robot rights might emerge. They mostly focus on qualitative discussion, hypothesizing, and forecasting. They expect increasing support for robot rights. Non-European-originating philosophical perspectives are considered
Mehlman et al. (2017)	The authors discuss the rights and responsibilities of “artificially intelligent robots.” The capacity for “pain and suffering,” self-awareness, and “human-like traits” are treated as relevant criteria. The authors present these issues as in need of further research but seem open to the idea of rights for AIs
Michalski (2018)	Michalski argues that existing legal precedents are presented as poor fits because they do not meet various “basic ontological and deontological thresholds.” Therefore, a new legal category is encouraged for artificial persons, which could “lay the groundwork for a substantive status as a kind of person entitled to basic and constitutional rights”
Miles (1994)	This is a review of two novels, one of which deals with topics relating “rights for artificial intelligences” and whether they can be enslaved. The book reportedly “reminds us that… unless we begin preparing for this pretty soon, we are bound to be storing up trouble for ourselves”
Miller et al. (2015)	Miller, Wolf, and Grodzinsky “contend that sophisticated machines that can function for extended periods of time without direct human intervention (our definition of ‘intelligent and autonomous machines’) ought to be considered for designation as moral patients. Relatively simple devices that fit this description already exist.” Their evaluation of agency and patiency (which they treat as closely interlinked) draws on Floridi’s Information Ethics
Miller (2015)	Miller focuses on “maximally human-like automata as test cases for a theory of moral status.” Many past contributions are critiqued, including those that emphasise ontological criteria and social-relational approaches. Noting that automata are created by humans and that humans “discern, affirm, and thereby realize human rights,” Miller argues that, “within human rights institutions or in a liberal democracy one is not obliged to grant humanlike automata full human rights.” Nevertheless, Miller concedes that, “there may be some justification” for granting a limited set of rights to automata
Mosakas (2020)	Mosakas argues that “phenomenal consciousness,” which “underlies the morally relevant properties (such as sentience),” is “a necessary condition for accrediting moral status.” Mosakas concludes that, “social robots should not be regarded as proper objects of moral concern unless and until they become capable of having conscious experience. Various alternative approaches, such as those of Gunkel, Coeckelbergh, Floridi, and Tavani, are critiqued
Nakada (2011)	This article is focused on a mostly irrelevant topic. However, results from surveys in 2008 (n = 500) and 2010 (n = 431) asking about views on robots in Japan are reported. Only 9.4% and 13.5% agreed with the statement: “Robots should be given similar rights in the future as fetuses or patients in a coma without consciousness or awareness.” Additionally, 21.4% and 29.6% agreed with the statement: “Robots are expected to be a subject of affection or consideration in the future just as the earth, mountains, rivers are treated so, even though they have no life”
Nakada (2012)	This focuses on a similar topic to Nakada (2011). The same survey results are reported, plus responses to the same survey questions from China and Thailand in 2010 and 2012, respectively. Chinese respondents seemed most supportive of robot rights, followed by Thai respondents
Navajas et al. (2019)	This study presented participants “with a set of moral scenarios” and asked them “to judge the acceptability of a controversial action.” In smaller groups, participants “discussed their opinions to see whether they agreed on common values of acceptability.” One scenario asked whether it was acceptable to delete an AI that was “indistinguishable from human intelligence.” The authors do not report the results by scenario in the paper but the raw data is freely accessible for reanalysis
Neely (2014)	Neeley argues that “interests” is a more important criterion for the inclusion of intelligent machines in the “moral community” than sentience; sentience, self-awareness, and autonomy could all be sufficient. These features operate on spectrums that affect moral inclusion. The arguments could also apply to entities that are “not embodied and have only a virtual presence”
Nijssen et al. (2019)	The first experiment found that, “robot-like robots were sacrificed significantly more often than humans and humanlike robots,” though “the effect of humanized priming was the same for all three agent types.” The second experiment suggested that the attribution of “affective states” had a larger effect on willingness to sacrifice entities than agency
Nomura et al. (2018)	In this study, Japanese students interacted with a robot and were asked survey questions. The results suggest that, “the participants having higher expectation of rapport with a robot showed more moral expansiveness for the robot” and “[t]he robot’s relational behaviors… did not have effect on the participants’ rapport expectation of the robot”
Nomura et al. (2019)	This article describes the development of a “Moral Concern for Robots Scale (MCRS).” It includes adapted questions from several previous scales and was developed through a survey with 121 Japanese university students and a factor analysis on the results. It contains a subscale for “basic moral concern” for robots and another on “concern for psychological harm”
Nyholm (2019)	Nyholm sees “whether we should extend any kind of moral consideration to robots that actually exist already or that will exist in the near future” as a “more pressing question, with greater real-world relevance” than whether they will warrant moral consideration in the more distant future. Views of other contributors – Bryson, Gunkel, Coeckelbergh, Danaher, Darling and Richardson – are summarized
Obodiac (2012)	Robots are considered within a broader discussion of citizenship. Various theories of citizenship are employed, especially the Greek concept of nomos
Olivera-La Rosa (2018)	This article examines “the phenomenology of the uncanny feeling,” that people experience when encountering “human-like stimuli” such as humanoid robots. Relevant studies that have examined this are summarized and analyzed through a “social functionalist account.” Olivera-La Rosa hypothesizes that the uncanny feeling “constrains the ‘moral circle.’”
Pagallo (2010)	Pagallo notes several legal precedents that could apply to the rights and obligations of artificial entities: “robots as killers,” “robots as fridges,” “robots as slaves,” and “robots as pets.” Pagallo also draws on Floridi to argue that robots are “moral ‘patients ‘ or receivers that deserve respect and protection”
Pagallo (2011)	Pagallo notes several legal precedents that could apply to the rights and obligations of artificial entities: “robots as kids,” “robots as pets,” “robots as employees,” and “robots as slaves.” Like Floridi, Pagallo argues that “ ‘good’ or ‘evil’ can conveniently be represented as anything that enhances or damages the informational complexity of the whole” and comments that robots “are informational objects par excellence”
Petersen (2007)	Petersen outlines a philosophical argument in defense of “designing robots so that they want to serve (more or less particular) human ends”, but does not defend robot slavery, which is understood to mean “to be forced into work contrary to your will”
Petersen (2012)	After outlining some previous arguments on the ethics of robot servitude, Petersen notes their belief that, “it is possible to create robots of ethical significance” — “artificial people” that “could have full ethical standing like our own.” Nevertheless, as in their 2007 article, Petersen argues that designing robots so that “comply with our intentions for them to be our dedicated servants” could still be “ethical”
Piazza et al. (2014)	The authors argue that “harmfulness… is an equally if not more important determinant of moral standing” than moral “patiency” or “agency.” The authors’ studies are of low relevance, but they summarize relevant research, such as one paper where “participants rated a range of [13 different] natural and unnatural entities,” including a robot, “on attributes pertaining to what they labeled as ‘experience’… and ‘agency.’” These factors were found to be correlated
Powers (2013)	This is an article primarily about “moral agency of computers” with a brief discussion about moral patiency. Powers argues that agency and patiency are substantially separate and, contra Floridi, that “computers are unlikely to be moral patients”
Prescott (2017)	Prescott argues that the description of robots as tools “assumes, all too easily, that we know the boundary conditions of future robotics development” and that, “[t]he ontological status of robots might be best described as liminal – neither living nor simply mechanical.” Some psychological evidence is summarised. Prescott takes seriously the concern that, in the future, robots could “have some significant psychological capacities” but people would “still see them as just tools”
Puaschunder (2019)	Robots and AI are presented as becoming increasingly autonomous and challenging perceptions of consciousness and legal personhood, “gaining human or quasi-human status.” Some issues relating to death, killing, and suicide are discussed with occasional reference to AI
Rademeyer (2017)	After reviewing various previous writings relating to robot rights, Rademeyer uses a “causal layered analysis” to briefly explore three possible scenarios for the development of robot rights. Rademeyer suggests that the “equal rights” scenario would be the “preferable future.” Discussions of “rapid technological advancement” and predictions of the singularity are used to convey urgency
Rainey (2016)	“This paper asks whether and how an artefact, such as a robot, could be considered a citizen… Three key notions emerge in the discussion: discursivity, embodiment and recognition.” Rainey draws on Kant and Aristotle to argue that, “citizens must be a community, with a sense of community, and at least be disposed to taking an interest in the governance of that community”
Redan (2014)	“How would we treat a robot as intelligent and complex as ourselves? Ben Redan argues that the justifications used to grant all humans a special moral status would also apply to advanced artificial intelligence, and that it may be in our collective interest to treat such entities the way we would like to be treated by them”
Reiss (2020)	Reiss argues that, “given that robots are being made with ever greater powers of cognition, at some point these powers of cognition may reach the point at which we need to start talking of robots as having minds and being persons.” Evolutionary, chemistry, historical (moral circle expansion) and theological perspectives are addressed briefly. Reiss encourages drawing comparisons to slavery and other precedents in moral education
Richardson (2016)	Richardson compares “the rights of machines” to slavery and other human rights issues, arguing that, “[e]xtending rights to machines has the potential to reduce the idea of what it means to be human”
Richardson (2019)	Various aspects of human–robot interaction are explored ”by reference to the work of Martin Buber in I and Thou.“ Richardson argues that robots are commodities whereas animals are not, so comparison between robots and animals is unhelpful
Risse (2019)	Risse Distinguishes between the ”medium term“ where machines do not yet ”come up for moral consideration,“ and the ”long term” where they plausibly will have moral status, even if they are not conscious. Theories of consciousness are discussed
Robertson (2014)	Robertson discusses and analyzes elements of Japanese culture that relate to robot rights. The example of the treatment of Paro, the care robot, is discussed
Rodogno (2017)	This chapter argues that, “as long as robots lack certain features, they can be neither moral agents nor moral patients.” Previous contributions are critiqued and the “psychological and biological bases” for seeing current artificial entities as moral agents or patients are considered
Russell (2009)	Russell discusses the potential rights of sex robots. Russell argues that humanoid robots could become conscious in the future “and therefore deserving of ‘intrinsic rights’ far beyond the legal personhood status for regulatory purposes.” Some potential legal pathways to granting rights to robots are discussed. Sex with robots is seen as a potential catalyst
Sætra (2019)	Sætra discusses several possible criteria for demarcating animals or AI from humans or for granting them moral consideration, including “reason,” the capacities for science and language, consciousness, emotions, and moral capacity. These are critiqued and Sætra notes that, “it is increasingly difficult for us to place a philosophically sound border between us and them”
San José et al. (2016)	The authors assume that, in the future, superintelligent AI “will have consciousness” and perhaps “an even higher moral status than humans.” They explore philosophical issues if this assumption is taken, using “Kant’s definition of consciousness and variations of utilitarianism”
Sarathy et al. (2019)	The authors discuss “consent” as ”a distinct, critical area for HRI research.” There is some brief discussion of abuse of robots. They note that verbal abuse towards machines might not matter morally, apart from insofar as it “sets the wrong normative tone in the environment” and may have negative indirect effects on observers
Schafer (2016)	Schafer analyzes the European Parliament’s Resolution that relates to electronic personhood and discusses it through comparison and analogy to science fiction writings
Scheessele (2018)	The article argues that, “some current and foreseeable intelligent machines have approximately as much moral status as plants, trees, and other environmental entities… the upper limit of our obligations should not exceed the upper limit of our obligations toward plants, trees, and other environmental entities.” “Moral agency” is seen as a key criterion and consciousness is not presented as required for an entity to have “a good of its own”
Schmetkamp (2020)	Schmetkamp argues that we can have empathy for social robots. Schmetkamp discusses several adjacent moral issues and notes that, “robots do not actually feel or experience anything,” but does not focus specifically on moral status
Schwitzgebel and Garza (2015)	Schwitzgebel and Garza argue that “artificially intelligent beings” could exist that would deserve moral consideration, especially if they were created by humans. They argue that, “there must be some relevant difference between the two entities that grounds [any] difference in moral status.” They defend this argument against possible Kantian, utilitarian, Aristotelian and social-relational objections. They make some recommendations for AI design to reflect this
Sheliazhenko (2019)	This short article focuses on the description of a “[m]odel program of judge autonomy… written in Java programming language.” The abstract argues for immediate increases in rights for robots, but the reasoning is unclear and the article does not seem to elaborate on this
Sijie (2020)	Sijie uses an “ant colony optimization” algorithim to, “improve the path planning ability of the intelligent robot, and it is simulated to analyze the personality rights of the intelligent robot.” Based on the findings, Sijie argues that robots will inevitably given personality rights as they become more intelligent
Siponen (2004)	Floridi’s Information Ethics is summarized and critiqued. Siponen argues, for example, that Information Ethics is “too all-embracing, if the killing of an insect (e.g., a cockroach) is morally wrong in every case”
Sittler (2018)	This paper discusses “[t]he expected value of the long-term future.” Though not referring directly to artificial sentience, it discusses risk factors for suffering of artificial entities and actions that could be taken to address them. In citing Sotala and Gloor (2017) and using phrases such as “powerless sentient beings,” it seems likely that the author is intending to consider the implications for artificial sentience
Smids (2020)	Smids offers four criticisms of Danaher’s (2020) theory of ethical behaviorism. For example, one critique is that, “Ontology Matters (a Lot)” and “inner” states are crucial. Smids sees “sentience and cognitive capacities” as the “properties that may ultimately ground moral status” and “rights” and “duties” are only discussed through direct quotes
Sommer et al. (2019)	In this study, “Children (4–10 years of age, N = 126) watched videos of six different entities having a box placed over them that was subsequently struck by a human hand. The results suggest that, ”[c]hildren afforded robotic entities less moral concern than living entities but afforded them more moral concern than nonliving entities, and these effects became more pronounced with age.“ Correlates and predictors are examined
Sotala and Gloor (2017)	Sotala and Gloor summarize three “pathways that could lead to the instantiation of large numbers of suffering subroutines” in the future: “Anthropocentrism,” “[i]ndifference” to human values, or “[u]ncooperativeness” to other moral frameworks. They also note that, ”simulations of sentient beings“ might exist and suffer on vast scales. This is presented as a clearly negative outcome and risks recommendations are made for actions to reduce risks of astronomical future suffering
Sparrow (2004)	Sparrow proposes a test for when machines have achieved “moral standing comparable to a human” – when replacing a human with a machine in a moral dilemma “leaves the character of the dilemma intact.” Sparrow argues that, though machines’ cognitive abilities will increase, they will not achieve full “personhood,” which also implies “an interdependent network of moral and affective responses, such as remorse, grief and sympathy,” unless they are given human form
Sparrow (2012)	As in their 2004 paper, Sparrow proposes a test for when machines have achieved “moral standing comparable to a human” – when replacing a human with a machine in a moral dilemma “leaves the character of the dilemma intact.” This is about more than just extending the concept of *Homo sapiens,* which might imply that AI would take moral priority over humans
Sparrow (2020)	Sparrow argues that, “[e]ven if an agent’s ‘cruel’ treatment of a robot has no implications for their future behaviour towards people or animals… [it] gives us reason to criticise their actions. Viciousness towards robots is real viciousness.” However, “[o]nly in the rare case where it might be reasonable for someone to mistake a robot for the thing that it represents might spontaneous expressions of emotion and concomitant actions be virtuous”
Spence (2012)	Spence evaluates Floridi’s Information Ethics. Spence argues that ethical value comes from “purposive agency” rather than status as an informational object. Hence, non-sentient artificial entities may have moral value “inherently but conditionally by rational design,” whereas sentient beings have moral value “intrinsically and unconditionally”
Spence et al. (2018)	Spence, Edwards, and Edwards examined “willingness to sign a petition urging the United Nations to form a working group on the issue,” with participants being sent the request either by “a human confederate or Softbank’s Pepper humanoid robot.” 46% (n = 78) were willing to sign the petition. Using multiple regression, they found that,“[n]egative attitudes toward robots, prior experience interacting with robots, and perceived credibility of the petitioning agent were significant predictors”
Spennemann (2007)	Spennemann focuses on “cultural heritage” rights and argues that the justifications for granting rights to “great apes” might also apply to “future AI-driven robots” that “have the ability to make reflective decisions.” This would necessitate “processes that ensure that these rights are not violated when we are dealing with robotic heritage places and artefacts”
Stapleton (2018)	Stapleton focuses mostly on moral agency, but also briefly discusses patiency, noting that, “animals can be moral patients because they are sentient,” whereas “[r]obots may never be truly sentient and may never be designed to suffer (or even have emotions).” Intuitively, we might also feel that “a high level of intelligence” is sufficient for moral patiency, but if we use this criteria for robots it should apply to networks of animals, plants, or fungi too
Starmans and Friedman (2016)	Starmans and Friedman found that their study participants “were less likely to judge that [an] entity was owned when it was described as autonomous… and this pattern held regardless of whether the entity was a human or an alien (Experiments 1 and 3), a robot (Experiments 2 and 3), or a human-like biological creation (Experiment 2).” They also found that participants were “less likely to judge that humans are owned compared with other kinds of entities”
Sullins (2005)	This paper considers a number of different ethical questions relating to “artificial life.” Sullins briefly argues that artificial life should be granted moral consideration in the future if their cognitive capacities advance sufficiently, but could also warrant some moral consideration for similar reasons that environmental ethics grants ecosystems some moral consideration
Sumantri (2019)	Sumantri discusses various paths to regulating robots, noting, for example, that, “[i]f Indonesia follows in Saudi Arabia’s footsteps, then the responsibility will be borne by the AI robot as a citizen. The robot will have the right to sue and be sued” and be treated similarly to humans. The precedent of animals is also considered. Human interests are centred. No clear recommendations are made, though the issue is presented as urgent
Summers (2016)	Summers examines citizenship, voting rights, and other issues for AI and digital human minds. Summers predicts a “blurring lines between human and machine, introducing an environment in which machines are seen as being persons.” These topics are discussed with reference to science fiction films and “Marin Heidegger’s Philosophy of Technology”
Suzuki et al. (2015)	Suzuki et al. “performed electroencephalography in 15 healthy adults who observed either human- or robot-hand pictures in painful or non-painful situations such as a finger cut by a knife.” Their results suggest that, “we empathize with humanoid robots in late top-down processing similarly to human others. However, the beginning of the top-down process of empathy is weaker for robots than for humans”
Swiderska and Küster (2018)	This online study with 217 US participants found that, “both robotic and human-like avatars were imbued with mind to a higher degree” when they were presented with a facial wound, “irrespective of the baseline level of mind attributed to their unharmed counterparts”
Swiderska and Küster (2020)	Across several experiments, Swiderska and Küster found that, “[h]armful robotic agents were consistently imbued with mental states to a lower degree than benevolent agents” and that, “a human moral patient appeared to suffer less when depicted with a robotic agent than with another human. The findings suggest that future robots may become subject to humanlike dehumanization mechanisms, which challenges the established beliefs about anthropomorphism”
Taraban (2020)	Taraban focuses on the “emerging interdisciplinary field” of neurorobotics. A few news stories relating to the “Rights of Intelligent Robots” (such as the robot Sophia being granted citizenship) are summarized. Rhetorical questions are asked about robot rights. Taraban comments that robots may demand rights
Tavani (2008)	This article focuses primarily on the topic of information privacy. However, it begins with a summary of Floridi’s Information Ethics, including its approach to moral patiency
Tavani (2018)	Tavani begins with a thorough review of existing works on questions relating to whether social robots qualify for moral consideration. Tavani argues that the question of whether to grant rights to robots “is ambiguous and imprecise.” Tavani argues that humans may have “a direct moral duty” to nonhumans, including social robots, as part of “being-in-the-technological-world”
Theodorou (2020)	Noting the possibility for both moral agency and patiency of artificial entities, Theodorou argues that, “culture determines the moral status of all entities, as morality and law are human-made ‘fictions’ that help us guide our actions. This means that our moral spectrum can be altered to include machines. However, there are both descriptive and normative arguments for why such a move is not only avoidable, but also should be avoided.“
Toivakainen (2016)	This paper critiques Gunkel with reference to Levinas and several other theorists. The discussion of moral patiency is brief. Toivakainen argues that, unlike in the case of “living beings,” “the ethical responsibility in the case of artefacts is a responsibility towards ourselves and the kinds of persons we are”
Toivakainen (2018)	Toivakainen offers “brief critical remarks on the question of ‘robot rights,’” arguing that, “[a]lthough robots and other automation technologies are part of the dialectics of labor and equality, it is not the robots (themselves) that we need to think of in moral terms but rather the drive of instrumental reason behind it”
Tollon (2019)	This is a philosophy MA thesis, primarily focused on whether artificial entities can be moral agents, with some discussion of moral patiency. Tollon argues that, “machines may conceivably be moral patients in the future” and “there is a strong case to be made that they are (or will very soon be) moral agents”
Tollon (2020)	Tollon critiques he Organic View outlined by Torrance (2008). Following Coeckelbergh, Tollon prioritizes critiques reliance on “ontological features” of entities for decision-making about whether to grant them moral consideration. Noting that our intuitions about sentience may be incorrect, Tollon argues that we can also reasonably use “behavioral cues” and “other relevant social-relational criteria”
Tomasik (2011)	Tomasik argues that there could be “astronomical future suffering” through the “[s]pread of wild animals,” the running of “[s]entient simulations… that are sufficiently self-aware as to feel what we consider conscious pain,” the creation of “[s]uffering subroutines,” i.e. “certain algorithms” that “might be sufficiently similar to the pain programs in our own brains that we consider them to actually suffer,” or “[b]lack swans.” Given vast computing power, “digital suffering” may “vastly outweigh” biological suffering
Tomasik (2013)	This article, motivated to reduce the risk that artificial sentience suffers on an astronomical scale, discusses factors and interventions that could affect the risk of this happening, such as encouraging “[b]ig-picture, cosmopolitan thinking.” Tomasik argues that technical research in “[a]rtificial consciousness seems net harmful to advance,” but encourages philosophical dialogue
Tomasik (2014)	Tomasik argues that, “present-day artificial RL [reinforcement learning] agents have a very small but nonzero degree of ethical importance. This is particularly plausible for views according to which sentience comes in degrees based on the abilities and complexities of minds… [RL programs] may become more significant in the coming decades as RL is increasingly applied to industry, robotics, video games, and other areas”
Tonkens (2012)	Tonkens examines “whether the creation of virtuous autonomous machines is morally permitted” and argues that, “the creation of such machines violates certain tenets of virtue ethics, and hence that the creation and use of those machines is impermissible”
Torrance (2005)	Torrance considers moral agency and patiency for machines, with sentience and rationality as relevant criteria. One “robust response” would be that machines will never warrant “moral respect” unless they are “organisms.” But Torrance concludes that, “even if (non-organic) machines never achieve a fundamental moral status equivalent to that of humans… it looks as though there will nevertheless be many ways in which machines will be seen as fit holders of kinds of moral status”
Torrance (2006)	Torrance notes that, under some views, consciousness might be “a strict requirement for full moral status” and that this is “arguably, a remote possibility for electronic beings.” However, “there are still important ways in which non-conscious artificial agents could come to have moral responsibilities, and even rights, of sorts,” such as through property ownership
Torrance (2008)	This paper is primarily focused on assessing whether artificial entities could ever be sentient. There is some discussion of wider societal and ethical implications. The “organic view” is defined, where “artificial humanoid agents” cannot count as moral agents or “appropriate targets of intrinsic moral concern” because they will not be sentient or have sufficient “empathic rationality.” Torrance does not argue that this view is correct, however
Torrance (2011)	This article discusses moral patiency and agency or artificial entities. As with Torrance’s (2013) article, approaches in machine ethics are explored through four categories: “anthropocentrism,” “infocentrism,” “biocentrism,” and “ecocentrism”
Torrance (2013)	This article discusses moral patiency and agency or artificial entities in relation to the context of Singer’s terminology of speciesism and the expanding moral circle. Though Singer emphasizes sentience, Torrance notes that this might not be a requirement for moral consideration. Approaches in machine ethics are explored through four categories: “anthropocentric,” “infocentric,” “biocentric,” and “ecocentric”
Torrance (2014)	Torrance contrasts “realist” and “social-relational” perspectives on “judgments of the moral status of artificial agents,” arguing in favor of the former and the importance of “conscious satisfaction or suffering.” Torrance accepts that determining consciousness is difficult but argues that it is still important. Torrance argues that current artificial entities do not have conscious satisfaction or suffering but that future artificial entities might
Torres (2018)	Torres argues that argues that space colonization is undesirable because it increases the risk of various forms of astronomical suffering. Torres evaluates strategies to reduce this risk. Torres briefly cites various Tomasik papers to note that, “someone somewhere” would run sentient simulations and “create new biospheres in which wild animals are subject to Darwinian misery”
Torres (2020)	Torres explores whether anti-natalists can oppose human extinction. In doing so, Torres discusses “whole-brain emulation” and “mind-uploading,” both of which would blur the distinction between humanity and artificial sentience. Torres notes uncertainty about whether and when these technologies will be developed, but seems to assume that “ems” or uploaded minds would warrant moral consideration, such as noting that terminating “ems” would constitute mind crime
Turchin (2019)	This paper argues that “A Computer Simulation of the Past Could be Used for Technological Resurrection,” which is seen to be a broadly positive development. Some ethical concerns, such as mind crime, are discussed, though the assumption of the paper seems to be that simulated lives have moral value
Turchin et al. (2019)	The authors explore “what is the most probable type of simulation in which humanity lives (if any) and how this affects simulation termination risks.” The article seems to assume that simulations could be morally important, since all known life could simply be simulated
Turner (2019)	Turner considers various legal issues relating to AI, including discussing AI and robot rights from both a legal and moral perspective. Turner gives four reasons why we might want to consider granting rights to robots: “(i) if they are conscious and can suffer; (ii) from compassion, if they appear to be conscious; (iii) their value to humans; and (iv) where humans and AI may are combined”
Tzafestas (2016)	Consciousness, “feelings,” and “interest” are seen as criteria for robot rights. Bryson’s, Kantian, and contractualist perspectives are summarized. This is a fairly brief discussion in a chapter summarizing various issues in roboethics
Umbrello and Sorgner (2019)	This paper focuses on the possibility of suffering in “nonconscious” artificial entities and whether they warrant moral consideration. Umbrello and Sorgner remain open to the possibility and encourage further research into how “nonconscious cognitive suffering… may be instantized in wetware developments in AI research”
Vadymovych (2017)	Vadymovych considers robot rights from the perspective of legal precedent. Vadymovych argues for legal personhood and rights for robots
Van den Berg (2011)	Van den Berg focuses broadly on “techno-regulation” – robot rights is considered only briefly. A few relevant contributions are summarized. Comparisons are made to the legal status of animals and other nonhuman entities
Van den Hoven Van Genderen (2018)	Van Genderen discusses the possibility of legal personhood rights for AI entities with relation to whether such entities will be able to become “legal actor[s] with legal capacity” and whether they are “natural persons.” The precedents of slaves, women, animals, and corporations are considered and several theories of personhood are summarized. AI personhood is more or less possible within these different conceptualizations of personhood
Vanman and Kappas (2019)	This is a review of social psychological research about “Social Robots for Intergroup Relations.” It notes, for example, that, “[p]eople tend to perceive social robots as autonomous and capable of having a mind… As social robots become more human like, people may also feel greater empathy for them,” but this can also “challenge our human distinctiveness, threaten our identity, and elicit suspicion”
Vize (2011)	Vize examines moral consideration of machines from a utilitarian perspective, drawing on Peter Singer, arguing that “sentience is both necessary and sufficient for moral considerability, and utilitarians must take care to avoid substratism.” Vize argues that, “because the methods we use to tell if another being is conscious are unreliable in the case of machines, then the proper attitude toward machine consciousness is agnosticism”
Voiculescu (2020)	Voiculescu analyzes documents from international organizations relating to legal and ethical issues “so that human rights standards are adapted to new conceptual and content challenges”
Wallkötter et al. (2020)	In two online experiments, the authors show that both framing of the robots as having high or low mind and manipulated descriptions of robots’ level of social engagement with humans “independently influence participants’ mind perception” of robots. “However, when we combined both variables in the following real-world experiment, these effects failed to replicate”
Wang and Krumhuber (2018)	In two experiments, the authors found that “robots with social function were perceived to possess greater ability for emotional experience, but not cognition, compared to those with economic function and whose function was not mentioned explicitly.” A further two experiments found that economic and social value affected ascriptions of cognitive and emotional capacity
Ward et al. (2013)	Ward, Olsen, and Wegner conducted four experiments and “found that observing intentional harm to an unconscious entity—a vegetative patient, a robot, or a corpse—leads to augmented attribution of mind to that entity. A fifth experiment reconciled these results with extant research on dehumanization by showing that observing the victimization of conscious entities leads to reduced attribution of mind to those entities”
Wareham (2013)	Wareham argues that consequentialist and “interest-based theories” of moral status “should be rejected, since they undermine the ideal that persons are moral equals.” In contrast, “respect-based theories,” “based on kantian and contractualist accounts,” are presented as more intuitive. This suggests that, “an artificial agent has moral status equal to that of a person if it has the capacity for reflective self-control.” Wareham argues that psychological testing could shed light on this
Warwick (2010)	Warwick examines the topic of “culturing neural tissue and embodying it in a mobile robot platform,” including the technology itself and several related ethical issues. Given that these robots could have “more brain cells than a cat, dog or chimpanzee,” Warwick suggests that, “[s]urely a human neuron robot must have [the same rights as animals] and more?”
Warwick (2012)	As in their 2010 paper, Warwick examines the idea of a robot with a biological brain, which could be developed soon. Given the biological brain, Searle’s Chinese Room argument against AI consciousness would not apply. Warwick asks: “If a robot body contains a brain of 100 billion human neurons then should that robot be afforded the same rights as a human?”
Waser (2012)	Waser argues that “we need to stop instinctively and reflexively acting on our different evolved ratchets and work together fleshing out our top-down design and justifications until everyone can accept it as moral.” Waser sees one implication of this as being that “Safety and Morality REQUIRE the Recognition of Self-Improving Machines as Moral/Justice Patients and Agents”
Wegloop and Vach (2020)	The authors argue that it is not possible to simulate consciousness then briefly explore several ethical implications of this, such as that there is no ethical difference between simulating a suffering human or a happy human. However, they note that their arguments do not show that “robots do not suffer” and suggest it might sometimes still be best to behave as if simulations were conscious
Weng et al. (2009)	The authors consider legal precedent for the treatment of robots, including a brief section on robot rights. They argue that, if we take certain legal pathways, “we may need to spell out robot rights and responsibilities in the same manner that we do for such non-human entities as corporations”
Winsby (2013)	Assuming that, “pain has a certain (unpleasant) character” and that, “beings that can feel pain are owed some level of moral consideration,” Winsby argues that “pain engineering in AI is prima facie morally wrong”
Wortham (2018)	Through surveys on Amazon Mechanical Turk, Wortham finds that, “the addition of a visual or vocalised representation of the internal processing and state of a robot significantly improves the ability of a naive observer to form an accurate model of a robot’s capabilities, intentions and purpose.” Additionally, “A zoomorphic robot is perceived as more intelligent and more likeable than a very similar mechanomorphic robot.“
Wright (2019)	Wright argues that sentience and self-awareness are insufficient to grant constitutional rights to “advanced robots”; “both objective and subjectively adopted interests” are also required. Wright argues that granting rights excessively to entities without such “interests” could cause “avoidable net suffering” to other entities, though artificial entities need not be equal to humans to warrant rights.. Various other specific rights and risks are considered
Wu (2012)	Wu discusses rights relating to “machine speech.” The legal precedent of animals and corporations is considered. The usage of the US Constitution’s First Amendment is examined in detail. Wu argues that “merely functional” speech and speech by tools doesn’t tend to be granted First Amendment protection. Some artificially created “speech product[s]” can be protected
Wurah (2017)	Wurah looks at legal personality for robots through comparison to corporations, animals, and human rights theories. Whereas animal rights are conceptualized in terms of protecting the animals, “electronic personality” is conceptualized more in terms of protecting humans. Wurah remains neutral on the question of whether robots should be granted rights or not
Yampolskiy (2013)	Yampolskiy focuses mostly on protecting humanity against artificial general intelligence. Robot rights and other aspects of robot ethics are seen as distractions from more important topics. Yampolskiy argues that “machines should be inferior by design; they should have no rights and should be expendable as needed… since machines can’t feel pain”
Yampolskiy (2017)	Yampolskiy argues that, “computers are… at least rudimentarily conscious with potential to eventually reach superconsciousness” and proposes “a test for confirming certain subjective experiences.” This consciousness is seen to make artificial entities (including simulations) potential rights holders and moral patients
Yanke (2020)	Looking at legal precedent for modification of marriage laws in the US, Yanke argues that robots will have to possess sentience and autonomy to marry, but that, “it is social acceptance… rather than personhood criteria” will most influence legal development. Citing Singer, Yanke argues that, “AIs with specific human-like qualities cannot be justifiably denied certain rights”
Zenor (2018)	Zenor “suggests that an AI movement would parallel other civil rights movements and examines what legal doctrines could support legal personhood for artificial intelligence”
Ziesche and Yampolskiy (2018)	Ziesche and Yampolskiy focus on the interests of artificial sentience “to avoid suffering and to have the freedom of choice about their deletion.” Sentience is seen as the key criteria for moral patiency. Several plausible future outcomes for artificial sentience are considered. They suggest the creation of “the new field of AI welfare science” which would contribute both to “antispeciesism” and “AI safety”
Ziesche and Yampolskiy (2019)	Ziesche and Yampolskiy argue that, in order to transfer human minds to “other substrates… numerous other potentially sentient beings will have to be created.” They analyze “the additional suffering and mind crimes that these scenarios might entail.” They suggest creating indicators of artificial suffering. Tomasik and Bostrom are cited as arguing that artificial sentience may come to exist and may have “moral status”

**Table 7:** Categorization and scoring of search results

References	Search terms/identified how	Search term categories	Discipline	Country of institution	Argues for moral consideration?	Primary framework or moral schema used	Google Scholar citations
Abdullah (2018)	Google Scholar: 1	Rights	Other humanities	Malaysia	3.5	Deontological	0
Adam (2008)	Google Scholar: 7 and ACM Digital Library: 5 and 7	Moral	Other social sciences	United Kingdom	NA	NA	36
Akechi et al. (2018)	Scopus: 5 and Web of Science: 5	Moral	Cognitive science	Japan	NA	NA	6
Al-Fedaghi (2007)	Google Scholar: 5	Moral	Computer engineering or computer science	Kuwait	4.5	Information Ethics	3
Allen and Widdison (1996)	Google Scholar: 4	Rights	Law	United Kingdom	4	Legal precedent	224
Anderson (2012)	Google Scholar: 5 and 7 and Scopus: 7	Moral	Philosophy or ethics	United States	NA	NA	5
Andreotta (2020)	Google Scholar: 1, Scopus: 5 and Web of Science: 5	Rights/Moral	Philosophy or ethics	Australia	4	Mixture (deontological, consequentialist)	0
Armstrong et al. (2012)	Google Scholar: 9 and ACM Digital Library: 9	Suffering	Other or unidentifiable	United Kingdom	4	NA	85
Arnold and Gough (2017)	Google Scholar: 2	Rights	Law	Australia	NA	NA	0
Asaro (2001)	ACM Digital Library: 1	Rights	Philosophy or ethics/Computer engineering or computer science	United States	NA	NA	0
Asekhauno and Osemwegie (2019)	Google Scholar: 4	Rights	English literature or language/Other humanities	Iran	3.5	Unclear	0
Ashrafian (2015a)	Google Scholar: 5	Moral	Medical or biology	United Kingdom	4	Unclear	36
Ashrafian (2015b)	Google Scholar: 1	Rights	Medical or biology	United Kingdom	NA	NA	43
Barfield (2015)	Google Scholar: 2	Rights	Other engineering	United States	4	Legal precedent	0
Barfield (2018)	Google Scholar: 2	Rights	Other engineering	United States	NA	Legal precedent	15
Bartneck and Keijsers (2020)	Google Scholar: 1	Rights	Other social sciences	New Zealand	NA	NA	0
Basl (2013a)	Google Scholar: 7	Moral	Philosophy or ethics	United States	4	Mixture (consequentialist, deontological)	7
Basl (2013b)	Google Scholar: 7	Moral	Philosophy or ethics	United States	4	Mixture (consequentialist, deontological)	0
Basl (2014)	Google Scholar: 7 and Scopus: 7	Moral	Philosophy or ethics	United States	4	Mixture (consequentialist, deontological)	18
Beckers (2018)	Google Scholar: 10	Suffering	Philosophy or ethics	Netherlands	4.5	Consequentialist	0
Belk (2018)	Google Scholar: 1 and 3 and Scopus: 1	Rights	Business	Canada	4	Unclear	13
Bennett and Daly (2020)	Google Scholar: 2 and Scopus: 1 and 2	Rights	Law	Australia	NA	Legal precedent	0
Beno (2019)	Google Scholar: 1 and 2 and Scopus: 1	Rights	Other or unidentifiable	Slovakia	NA	NA	1
Bess (2018)	Google Scholar: 3, Scopus: 3, and Web of Science: 3	Rights	History	United States	4	Mixture (deontological, virtue ethicist, consequentialist)	3
Bigman et al. (2019)	Google Scholar: 1 and 6, ScienceDirect: 1 and 5 and 6, Scopus: 1, and Web of Science: 1	Rights/Moral	Psychology/Cognitive science	United States	NA	NA	28
Biondi (2019)	Google Scholar: 5	Moral	Philosophy or ethics	United States	4.5	Mixture (consequentialist, deontological)	0
Birhane and van Dijk (2020)	Google Scholar: 1, ACM Digital Library: 1, and Scopus: 1	Rights	Computer engineering or computer science	Ireland	1	NA	5
Birmingham (2008)	Scopus: 5	Moral	Computer engineering or computer science	United States	1.5	Deontological	0
Bolonkin (2012)	ScienceDirect: 1 and 2	Rights	Other or unidentifiable	United States	NA	NA	21
Bostrom (2014)	Google Scholar: 9	Suffering	Philosophy or ethics	United Kingdom	4	Consequentialist	2513
Bostrom et al. (2016)	Google Scholar: 9	Suffering	Philosophy or ethics	United Kingdom	4	NA	9
Brey and Søraker (2009)	ScienceDirect: 5 and 7	Moral	Philosophy or ethics	Netherlands	NA	NA	62
Briggs (2015)	Google Scholar: 7	Moral	Other social sciences/Computer engineering or computer science	United States	NA	NA	0
Briggs et al. (2014)	Google Scholar: 7	Moral	Other social sciences/Computer engineering or computer science	United States	NA	NA	5
Broman and Finckenberg-Broman (2018)	Google Scholar: 2	Rights	Law	Australia	4.5	Legal precedent	0
Bryson (2012)	Google Scholar: 7	Moral	Computer engineering or computer science	United Kingdom	1	Consequentialist	5
Bryson (2018)	Google Scholar: 1 and 7, ACM Digital Library: 7, Scopus: 7, and Web of Science: 7	Rights/Moral	Computer engineering or computer science	United Kingdom	1	Consequentialist	45
Bryson et al. (2017)	ACM Digital Library: 7	Moral	Computer engineering or computer science	United Kingdom	1.5	Mixture (legal precedent, consequentialist)	103
Calo (2016)	Google Scholar: 2	Rights	Law	United States	NA	Legal precedent	48
Calverley (2011)	Google Scholar: 3 and Scopus: 3	Rights	Law	United States	4	Mixture (legal precedent, consequentialist, deontological)	15
Cappuccio et al. (2020)	Google Scholar: 5 and 7 and Scopus: 5 and 7	Moral	Other engineering/information technology	Australia	4.5	Mixture (virtue ethicist, social-relational)	3
Cave et al. (2019)	Google Scholar: 7	Moral	Philosophy or ethics	United Kingdom	4.5	Deontological	6
Celotto (2019)	Google Scholar: 3, Scopus: 3, and Web of Science: 3	Rights	Law	Italy	NA	Unclear	1
Čerka et al. (2017)	ScienceDirect: 2	Rights	Law	Lithuania	NA	Legal precedent	41
Chernyak and Gary (2016)	Google Scholar: 5 and 6 and Scopus: 5	Moral	Psychology/Cognitive science	United States	NA	NA	11
Chesterman (2020)	Google Scholar: 2	Rights	Law	Singapore	3	Legal precedent	0
Chinen (2016)	Google Scholar: 7	Moral	Law	United States	NA	Legal precedent	13
Chomanski (2019)	Google Scholar: 6	Moral	Philosophy or ethics	United States	3.5	Virtue ethicist	3
Chopra (2010)	Google Scholar: 2 and ACM Digital Library: 2	Rights	Philosophy or ethics	United States	3	NA	16
Church (2019)	Google Scholar: 3	Rights	Medical or biology	United States	NA	NA	17
Coeckelbergh (2010a)	Google Scholar: 5 and 7 and ACM Digital Library: 5 and 7	Moral	Philosophy or ethics	Netherlands	4	Social-relational	95
Coeckelbergh (2010b)	Google Scholar: 1, 5, and 7, ACM Digital Library: 1, 5, and 7, Scopus: 1 and 5, and Web of Science: 1 and 5	Rights/Moral	Philosophy or ethics	Netherlands	3.5	Social-relational	135
Coeckelbergh (2013)	Google Scholar: 5 and 7 and ACM Digital Library: 5 and 7	Moral	Philosophy or ethics	Netherlands	NA	NA	2
Coeckelbergh (2014)	Google Scholar: 7	Moral	Philosophy or ethics	Netherlands	4	Social-relational	49
Coeckelbergh (2018)	Google Scholar: 7	Moral	Philosophy or ethics	Austria	3.5	Social-relational	4
Coeckelbergh (2020)	Google Scholar: 7	Moral	Philosophy or ethics	Austria	4	Social-relational	5
Craig et al. (2019)	Google Scholar: 1 and 2, ACM Digital Library: 1 and 2, and Scopus: 1	Rights	Communication or media/Robotics	United States	NA	NA	1
Dall’Agnol, Darlei (2020)	Google Scholar: 1	Rights	Philosophy or ethics	Brazil	4.5	Mixture (consequentialist, deontological, virtue ethicist, legal precedent)	0
Damholdt et al. (2020)	Google Scholar: 7	Moral	Medical or biology	Denmark	NA	NA	0
Danaher (2020)	Google Scholar: 5 and 6 and Scopus: 6	Moral	Law	Ireland	4.5	Other	17
Darling (2016)	Google Scholar: 1, 2, and 5	Rights/Moral	Communication or media	United States	4	Deontological (but similarities to the “social relational” approach)	122
Davidson et al. (2019)	Google Scholar: 5 and ACM Digital Library: 5	Moral	Psychology	Australia	NA	NA	0
Davies (2011)	ScienceDirect: 3	Rights	Law	United Kingdom	4.5	Legal precedent	54
Dawes (2020)	Google Scholar: 1	Rights	English literature or language	United States	NA	NA	0
De Graaf and Malle (2019)	Google Scholar: 1 and ACM Digital Library: 1	Rights	Computer engineering or computer science/Information technology	Netherlands	NA	NA	15
DiPaolo (2019)	Google Scholar: 1	Rights	Other social sciences	Canada	4.5	Unclear	0
Dixon (2015)	Google Scholar: 2	Rights	Other social sciences	Netherlands	4.5	Unclear	0
Dracopoulou (2003)	Google Scholar: 5	Moral	Medical or biology/other social sciences	United Kingdom	4	Mixture (consequentialist, deontological)	4
Drozdek (1994)	Google Scholar: 3	Rights	Computer engineering or computer science	United States	2	Mixture (virtue ethicist, deontological)	1
Drozdek (2017)	Google Scholar: 3	Rights	Computer engineering or computer science	United States	NA	NA	0
Erhardt and Mona (2016)	Google Scholar: 3	Rights	Law	Switzerland	4	Unclear	5
Estrada (2018)	Google Scholar: 1, ACM Digital Library: 1, and Scopus: 1	Rights	Philosophy or ethics	United States	4	Other	5
Estrada (2020)	Google Scholar: 6	Moral	Philosophy or ethics	United States	4	Other	1
Fagan (2019)	Scopus: 4	Rights	Law/Business	France	3	Legal precedent	0
Floridi (1999)	ACM Digital Library: 5	Moral	Philosophy or ethics	United Kingdom	4.5	Information Ethics	579
Floridi (2002)	ACM Digital Library: 5 and 7	Moral	Philosophy or ethics	Italy	4.5	Information Ethics	258
Floridi (2005)	ACM Digital Library: 5 and 7	Moral	Philosophy or ethics	Italy	4.5	Information Ethics	169
Fox (2018)	Google Scholar: 5 and 7	Moral	Philosophy or ethics	Netherlands	4	Social-relational	1
Frank and Nyholm (2017)	Google Scholar: 1 and 2 and 5 and ACM Digital Library: 1, 2, and 5	Rights/Moral	Philosophy or ethics	Netherlands	4	Mixture (deontological, consequentialist, social-relational)	35
Fraune et al. (2017)	Google Scholar: 5	Moral	Cognitive science	United States	NA	NA	13
Freier (2008)	ACM Digital Library: 5	Moral	Other or unidentifiable	United States	NA	NA	14
Friedman (2019)	Google Scholar: 1, 5, and 7	Moral	Philosophy or ethics	South Africa	4	Social-relational	0
Galanter (2020)	Google Scholar: 5	Moral	Design	United States	4.5	Mixture (other, deontological, consequentialist)	0
Gamez et al. (2020)	Google Scholar: 6, Scopus: 1, and Web of Science: 1	Rights/Moral	Philosophy or ethics	United States	4	Virtue ethicist	1
Gerdes (2015)	Scopus: 7	Moral	Design/Communication or media	Denmark	NA	NA	1
Gerdes (2016)	Google Scholar: 1 and 5 and ACM Digital Library: 1 and 5	Rights/Moral	Communication or media/Design	Denmark	1.5	Deontological	23
Gittinger (2019)	Google Scholar: 2	Rights	Philosophy or ethics	United States	NA	NA	0
Gloor (2016b)	Google Scholar: 8	Suffering	Other or unidentifiable	Germany	4.5	Mixture	5
Gordon (2020)	Google Scholar: 2, 3, and 7, Scopus: 3, and Web of Science: 3	Rights/Moral	Philosophy or ethics/Political science	Lithuania	4	Mixture (consequentialist, deontological)	8
Gray and Wegner (2012)	ScienceDirect: 2	Rights	Psychology	United States	NA	NA	294
Gregory (2012)	Google Scholar: 5	Moral	Philosophy or ethics	Australia	3	Mixture (consequentialist, deontological, virtue ethicist)	0
Gualeni (2020)	Google Scholar: 5, 6, and 7	Moral	Other or unidentifiable	Malta	4.5	Deontological	0
Gunkel (2007)	Google Scholar: 5, ACM Digital Library: 5 and 7, and Scopus: 5	Moral	Communication or media	United States	NA	Other	21
Gunkel (2012)	Google Scholar: 5 and 7 and ACM Digital Library: 5 and 7	Moral	Communication or media	United States	3	Other	239
Gunkel (2013)	Google Scholar: 5 and ACM Digital Library: 5	Moral	Communication or media	United States	NA	Social-relational	3
Gunkel (2014)	Google Scholar: 5 and 7 and Scopus: 5	Moral	Communication or media	United States	4	Social-relational	69
Gunkel (2015)	Google Scholar: 3 and 5, Scopus: 3, and Web of Science: 3	Rights/Moral	Communication or media	United States	3.5	Social-relational	13
Gunkel (2018a)	Google Scholar: 1 and 5 and Web of Science: 1	Rights/Moral	Communication or media	United States	4	Social-relational	93
Gunkel (2018b)	Google Scholar: 3 and 5	Rights/Moral	Communication or media	United States	4	Social-relational	3
Gunkel (2018c)	Google Scholar: 1, 5, and 7	Rights/Moral	Communication or media	United States	NA	Social-relational	51
Gunkel ( 2018d)	Scopus: 5	Moral	Communication or media	United States	4	Social-relational	0
Gunkel (2019a)	Google Scholar: 5	Moral	Communication or media	United States	4	Social-relational	2
Gunkel (2019b)	Google Scholar: 1	Rights	Communication or media	United States	4	Social-relational	0
Gunkel (2020a)	Google Scholar: 1, 5, and 6	Rights/Moral	Communication or media	United States	NA	NA	1
Gunkel (2020b)	Google Scholar: 1, 2, and 5	Rights/Moral	Communication or media	United States	4	Social-relational	0
Gunkel and Cripe (2014)	Google Scholar: 5	Moral	Communication or media	United States	4	Social-relational	1
Hagendorff (2020)	Google Scholar: 1, ACM Digital Library: 5, and Scopus: 5	Rights/Moral	Philosophy or ethics	Germany	4	Virtue ethicist	1
Hale (2009)	Google Scholar: 6	Moral	Philosophy or ethics	United States	4	Other	11
Hall (2005)	Scopus: 5	Moral	Computer engineering or computer science	United Kingdom	NA	NA	1
Hallqvist (2018)	Google Scholar: 2	Rights	Other social sciences	Sweden	NA	NA	0
Hanák (2019)	Google Scholar: 3 and 4	Rights	Philosophy or ethics	Czecia	2.5	Mixture (legal precedent, consequentialist, deontological)	0
Hartmann (2017)	Google Scholar: 6	Moral	Communication or media	Netherlands	NA	NA	24
Himma (2003)	Google Scholar: 7 and ACM Digital Library: 5 and 7	Moral	Philosophy or ethics	United States	4	Mixture (deontological, consequentialist)	25
Himma (2004)	ACM Digital Library: 5 and 7	Moral	Philosophy or ethics	United States	3	Mixture (deontological, consequentialist)	43
Hoffmann and Hahn (2020)	Scopus: 7 and Web of Science: 7	Moral	Other or unidentifiable	Switzerland	4	Social-relational	0
Hogan (2017)	Google Scholar: 5 and 7 and ACM Digital Library: 1, 5, and 7	Moral	Philosophy or ethics	United States	NA	NA	4
Holder et al. (2016)	Google Scholar: 2	Rights	Law	United Kingdom	NA	Legal precedent	20
Holm and Powell (2013)	ScienceDirect: 5	Moral	Philosophy or ethics/Communication or media	Denmark	NA	NA	12
Holy-Luczaj and Blok (2019)	Google Scholar: 6	Moral	Philosophy or ethics	Poland	4.5	Other	1
Hu (2018)	Google Scholar: 1	Rights	Law	United States	NA	Legal precedent	0
Hughes (2005)	Google Scholar: 2	Rights	Other or unidentifiable	United States	NA	NA	23
Huttunen et al. (2010)	Google Scholar: 1 and 2	Rights	Law	Finland	4.5	Legal precedent	12
Inayatullah (2001)	Google Scholar: 1	Rights	Future studies/Political science	Taiwan	4	Mixture (legal precedent, other)	3
Inyashkin (2016)	Google Scholar: 1	Rights	Other humanities	Russia	NA	NA	0
Jack et al. (2013)	Google Scholar: 5	Moral	Cognitive science	United States	NA	NA	70
Jackson Jr. (2018a)	Google Scholar: 9	Suffering	Other or unidentifiable	United States	3.5	Unclear	1
Jackson Jr. (2018b)	Google Scholar: 9	Suffering	Other or unidentifiable	United States	3	Unclear	7
Jackson and Williams (2019)	Google Scholar: 7	Moral	Computer engineering or computer science	United States	NA	NA	7
Jaynes (2020)	Google Scholar: 3 and 4, Scopus: 4, and Web of Science: 4	Rights	Other or unidentifiable	United States	5	Other (“capabilities-based altruism”)	3
Johnson and Miller (2008)	ACM Digital Library: 5	Moral	Philosophy or ethics	United States	2	Unclear	60
Johnson and Verdicchio (2018)	Google Scholar: 1 and 7, ACM Digital Library: 1, and 7	Rights/Moral	Philosophy or ethics	United States	1.5	Mixture (consequentialist, deontological, legal precedent)	9
Jowitt (2020)	Google Scholar: 4	Rights	Law	United Kingdom	4.0	Mixture (deontological, legal precedent)	0
Kaminska (2016)	Google Scholar: 2	Rights	Law	Netherlands	4	Unclear	0
Kaufman (1994)	Google Scholar: 5 and Web of Science: 5	Moral	Philosophy or ethics	United States	3	Other	12
Kelley and Atreides (2020)	Google Scholar: Google Scholar: 3 and ScienceDirect: 3	Rights	Robotics	United States	4.5	NA	1
Khoury (2016)	Google Scholar: 1 and 2	Rights	Law	Israel	1	Legal precedent	26
Kim and Petrina (2006)	Google Scholar: 1, 2, and 3	Rights	Other social sciences	United States	NA	NA	2
Kiršienė and Amilevičius (2020)	Google Scholar: 2	Rights	Law	Lithuania	3.5	Legal precedent	0
Klein (2016)	Google Scholar: 2 and 5, ACM Digital Library: 5 and 7	Rights/Moral	Communication or media	China	4	Consequentialist	4
Klein (2019)	Google Scholar: 5	Moral	Communication or media	China	4	Consequentialist	0
Klein and Lin (2018)	ACM Digital Library: 5	Moral	Communication or media	China	4	Unclear	2
Kljajić (2019)	Google Scholar: 8	Suffering	Philosophy or ethics	Croatia	3.5	Mixture	0
Kolling et al. (2016)	ScienceDirect: 5	Moral	Psychology	Germany	NA	NA	7
Kovic (2020)	Google Scholar: 10	Suffering	Other or unidentifiable	Switzerland	4	NA	0
Krämer (2020)	Google Scholar: 2	Rights	Computer engineering or computer science	Germany	NA	NA	0
Krebs (2006)	Google Scholar: 2	Rights	History	Germany	NA	NA	18
Kunnari (2020)	Google Scholar: 7	Moral	English literature or language	Finland	NA	NA	0
Kuran (2020)	Google Scholar: 5 and 7	Moral	Other or unidentifiable	Unclear	4	Mixture (deontological, consequentialist)	0
Küster and Świderska (2016)	Google Scholar: 5 and 7 and Scopus: 5 and 7	Moral	Psychology	Germany	NA	NA	3
Küster and Swiderska (2020)	Google Scholar: 5 and 7	Moral	Computer engineering or computer science	Germany	NA	NA	0
Küster et al. (2020)	Google Scholar: 7	Moral	Computer engineering or computer science	Germany	NA	NA	1
Laukyte (2017)	ACM Digital Library: 5	Moral	Law	Spain	4	Legal precedent	9
Laukyte (2019)	Google Scholar: 5	Moral	Law	Spain	4.5	Other	0
Laukyte (2020)	Google Scholar: 1	Rights	Law	Spain	NA	Legal precedent	1
Laulhe-Shaelou (2019)	Google Scholar: 1	Rights	Law	Cyprus	4	Mixture (consequentialist, social-relational, legal precedent)	0
Lavi (2019)	Google Scholar: 1 and 7	Rights/Moral	Design	Israel	3	Mixture (consequentialist, deontological)	0
Lee et al. (2019)	ACM Digital Library: 7	Moral	Psychology/Other engineering	Netherlands	NA	NA	1
Leenes and Lucivero (2014)	Google Scholar: 2	Rights	Law	Netherlands	NA	Legal precedent	71
Lender (2016)	Google Scholar: 7	Moral	Other or unidentifiable	Unclear	4.5	Consequentialist	0
Levy (2009)	Google Scholar: 1 and 5, Scopus: 1, and Web of Science: 1	Rights/Moral	Other or unidentifiable	United Kingdom	5	Social-relational	79
Levy (2012)	Google Scholar: 1 and 5 and ACM Digital Library: 5	Rights/Moral	Other or unidentifiable	United Kingdom	4.5	Legal precedent	34
Levy (2016)	Google Scholar: 1	Rights	Other or unidentifiable	United Kingdom	4.5	Legal precedent	10
Lima et al. (2019)	Google Scholar: 1 and 2	Rights	Computer engineering or computer science	Korea	NA	NA	0
Lima et al. (2020)	Google Scholar: 1, 2, and 5	Rights/Moral	Computer engineering or computer science	Korea	NA	NA	0
Loh (2019)	Google Scholar: 1, 5, and 7	Rights/Moral	Philosophy or ethics	Austria	4.5	Other	0
Lopez-Mobilia (2011)	Google Scholar: 5	Moral	Psychology	United States	NA	NA	0
Lupetti et al. (2019)	Google Scholar: 1	Rights	Design	Netherlands	3.5	Social-relational	3
MacDorman and Cowley (2006)	Google Scholar: 5	Moral	Other social sciences/Computer engineering or computer science	United States	4	Unclear	39
Mackenzie (2014)	ACM Digital Library: 5	Moral	Law	United Kingdom	4.5	Deontological	9
Mackenzie (2016)	Google Scholar: 6 and 7	Moral	Law	United Kingdom	4	Deontological	1
Mackenzie (2018)	Scopus: 1 and Web of Science: 1	Rights	Law	United Kingdom	4.5	Deontological	5
Mackenzie (2020a)	Google Scholar: 6	Moral	Law	United Kingdom	4	Other	0
Mackenzie (2020b)	Google Scholar: 6, ACM Digital Library: 6 and 7, and Scopus: 6	Moral	Law	United Kingdom	4	Deontological	8
Magnani (2005)	Google Scholar: 7	Moral	Philosophy or ethics	Itay	4.5	Deontological	1
Magnani (2007)	Google Scholar: 7	Moral	Philosophy or ethics	Itay	4.5	Deontological	0
Malle (2016)	Google Scholar: 1 and 5 and ACM Digital Library: 1 and 5	Rights/Moral	Cognitive science	United States	4	NA	78
Massaro and Norton (2018)	Google Scholar: 4	Rights	Law	United States	2.5	Legal precedent	0
Massaro (2015)	Google Scholar: 4	Rights	Law	United States	NA	Legal precedent	55
Mazarian (2019)	Google Scholar: 4 and 5	Rights/Moral	Cognitive science	Iran	1.5	Unclear	0
McLaughlin and Rose (2018)	Google Scholar: 1 and 7	Rights/Moral	Philosophy or ethics	United States	2.5	Mixture (deontological, consequentialist)	1
McNally and Inayatullah (1988)	Google Scholar: 1 and 2, ScienceDirect: 1 and ScienceDirect: 2	Rights	Law	United States	4	Mixture (legal precedent, other)	72
Mehlman et al. (2017)	Google Scholar: 2	Rights	Law	United States	4	NA	4
Michalski (2018)	Google Scholar: 2	Rights	Law	United States	4	Legal precedent	9
Miles (1994)	ScienceDirect: 4	Rights	Other social sciences	United Kingdom	NA	NA	3
Miller et al. (2015)	Google Scholar: 7	Moral	Computer engineering or computer science	United States	4.5	Information Ethics	10
Miller (2015)	Google Scholar: 3 and 7	Rights/Moral	Philosophy or ethics	United States	2	Deontological	14
Mosakas (2020)	Google Scholar: 5 and 6 and 7 and Web of Science: 3 and 5	Rights/Moral	Philosophy or ethics	Lithuania	4	Mixture (consequentialist, deontological)	0
Nakada (2011)	Google Scholar: 7	Moral	Other humanities/Other social sciences	Japan	NA	NA	0
Nakada (2012)	Google Scholar: 2	Rights	Other humanities/Other social sciences	Japan	NA	NA	3
Navajas et al. (2019)	ScienceDirect: 1	Rights	Cognitive science/Business	Argentina	NA	NA	4
Neely (2014)	Google Scholar: 3, 5, and 7 and Scopus: 3 and 7	Rights/Moral	Philosophy or ethics	United States	4.5	Mixture (consequentialist, deontological)	24
Nijssen et al. (2019)	Google Scholar: 5 and 6	Moral	Other social science	Netherlands	NA	NA	14
Nomura et al. (2018)	Google Scholar: 6 and Scopus: 6	Moral	Communication or media	Japan	NA	NA	1
Nomura et al. (2019)	Google Scholar: 5 and 6, ACM Digital Library: 5 and 6, and Scopus: 5	Moral	Communication or media	Japan	3	NA	1
Nyholm (2019)	Google Scholar: 1 and 5	Rights/Moral	Philosophy or ethics	Netherlands	NA	NA	1
Obodiac (2012)	Google Scholar: 2	Rights	Other humanities	United States	NA	NA	0
Olivera-La Rosa (2018)	Google Scholar: 6 and ScienceDirect: 6 and 7	Moral	Psychology	Colombia	NA	NA	6
Pagallo (2010)	Google Scholar: 7	Moral	Law	Italy	4.5	Mixture (legal precedent, Information Ethics)	11
Pagallo (2011)	Google Scholar: 7 and ACM Digital Library: 7	Moral	Law	Italy	4.5	Mixture (legal precedent, Information Ethics)	22
Petersen (2007)	Google Scholar: 1, ACM Digital Library: 1, Scopus: 1, and Web of Science: 1	Rights	Philosophy or ethics	United States	3	Mixture (consequentialist, virtue ethicist)	42
Petersen (2012)	Google Scholar: 1 and 5 and ACM Digital Library: 5	Rights/Moral	Philosophy or ethics	United States	3	Mixture (consequentialist, deontological, virtue ethicist)	41
Piazza et al. (2014)	Google Scholar: 6 and ScienceDirect: 5, 6, and 7	Moral	Psychology	United States	NA	NA	45
Powers (2013)	Google Scholar: 7, Scopus: 7, and Web of Science: 7	Moral	Philosophy or ethics	United States	3	Unclear	22
Prescott (2017)	Google Scholar: 7	Moral	Psychology/Robotics	United Kingdom	4	Unclear	18
Puaschunder (2019)	Google Scholar: 1	Rights	Other social sciences	United States	NA	Unclear	11
Rademeyer (2017)	Google Scholar: 2	Rights	Future studies	Australia	5	NA	0
Rainey (2016)	ACM Digital Library: 1	Rights	Philosophy or ethics	United Kingdom	3.5	Mixture (virtue ethicist, deontological)	7
Redan (2014)	Google Scholar: 2 and 5	Rights/Moral	Other or unidentifiable	Unclear	4.5	Unclear	1
Reiss (2020)	Google Scholar: 5	Moral	Other social sciences	United Kingdom	4	NA	0
Richardson (2016)	Google Scholar: 1, 3, and 5	Rights/Moral	Philosophy or ethics	United Kingdom	2	NA	60
Richardson (2019)	Google Scholar: 3 -Kathrani, ACM Digital Library: 1, and 3	Rights	Philosophy or ethics	United Kingdom	2	Unclear	6
Risse (2019)	Google Scholar: 5	Moral	Philosophy or ethics	United States	4	NA	0
Robertson (2014)	Google Scholar: 1, Scopus: 1, and Web of Science: 1	Rights	Other social science/History	United States	NA	NA	62
Rodogno (2017)	Google Scholar: 5 and 7, Scopus: 7, and Web of Science: 7	Moral	Other humanities	Denmark	2.5	Unclear	11
Russell (2009)	ScienceDirect: 1, 2, and 4	Rights	Law	United States	4	Legal precedent	11
Sætra (2019)	Google Scholar: 5 and 7	Moral	Political science	Norway	3	Unclear	2
San José et al. (2016)	Google Scholar: 9	Suffering	Philosophy or ethics	Denmark	4	Mixture (consequentialist, deontological)	0
Sarathy et al. (2019)	ACM Digital Library: 1	Rights	Computer engineering or computer science/Cognitive Science	United States	NA	NA	1
Schafer (2016)	Google Scholar: 2 and 5	Rights/Moral	Law	United Kingdom	NA	NA	3
Scheessele (2018)	Google Scholar: 7 and ACM Digital Library: 5 and 7	Moral	Computer engineering or computer science/Psychology	United States	3.5	Mixture (consequentialist, deontological)	2
Schmetkamp (2020)	Google Scholar: 5 and 7, Scopus: 5, and Web of Science: 5	Moral	Philosophy or ethics	Switzerland	NA	Social-relational	0
Schwitzgebel and Garza (2015)	Google Scholar: 5 and 7	Moral	Philosophy or ethics	United States	4	Mixture (deontological, consequentialist, virtue ethicist, social-relational)	28
Sheliazhenko (2019)	Google Scholar: 1 and Scopus: 1	Rights	Law	Ukraine	5	Unclear	4
Sijie (2020)	Google Scholar: 3	Rights	Law	China	NA	NA	0
Siponen (2004)	ACM Digital Library: 5 and 7	Moral	Communication or media/Information technology	Finland	3	Mixture	49
Sittler (2018)	Google Scholar: 10	Suffering	Other or unidentifiable	United Kingdom	4	Consequentialist	1
Smids (2020)	Google Scholar: 1 and 6	Rights/Moral	Philosophy or ethics	Netherlands	4	Consequentialist	0
Sommer et al. (2019)	Google Scholar: 5 and 6, ScienceDirect: 5 and 6, Scopus: 5 and Web of Science: 5	Moral	Psychology	Australia	NA	NA	5
Sotala and Gloor (2017)	Google Scholar: 8, 9, and 10, Scopus: 10, and Web of Science: 10	Suffering	Other or unidentifiable	Germany	4.5	Mixture	23
Sparrow (2004)	Google Scholar: 5 and ACM Digital Library: 5	Moral	Philosophy or ethics	Australia	3	Mixture (consequentialist, deontological)	52
Sparrow (2012)	Google Scholar: 1 and 5 and ACM Digital Library: 5	Rights/Moral	Philosophy or ethics	Australia	3.5	Mixture (consequentialist, deontological)	32
Sparrow (2020)	Google Scholar: 5	Moral	Philosophy or ethics	Australia	4.5	Virtue ethicist	1
Spence (2012)	ACM Digital Library: 5 and 7	Moral	Philosophy or ethics	Netherlands	4.5	Other	1
Spence et al. (2018)	Google Scholar: 1, 2 and 7, ACM Digital Library: 1, 2, 5, and 7, and Scopus: 1 and 5	Rights/Moral	Communication or media/Robotics	United States	NA	NA	3
Spennemann (2007)	ScienceDirect: 2	Rights	Other or unidentifiable	Australia	4	Consequentialist	21
Stapleton (2018)	Google Scholar: 7	Moral	Philosophy or ethics	United States	3	Unclear	0
Starmans and Friedman (2016)	Google Scholar: 6 and ScienceDirect: 6 and 7	Moral	Psychology	United States	NA	NA	9
Sullins (2005)	ACM Digital Library: 5	Moral	Philosophy or ethics	United States	4	Unclear	28
Sumantri (2019)	Google Scholar: 1	Rights	Other or unidentifiable	Indonesia	NA	Legal precedent	1
Summers (2016)	Google Scholar: 2	Rights	Philosophy or ethics	United States	NA	NA	0
Suzuki et al. (2015)	Google Scholar: 5	Moral	Computer engineering or computer science	Japan	NA	NA	77
Swiderska and Küster (2018)	Google Scholar: 7	Moral	Psychology	Poland	NA	NA	6
Swiderska and Küster (2020)	Google Scholar: 7, Scopus: 7, and Web of Science: 7	Moral	Psychology	Poland	NA	NA	1
Taraban (2020)	Google Scholar: 1, Scopus: 1, and Web of Science: 1	Rights	Psychology	United States	NA	NA	0
Tavani (2008)	ACM Digital Library: 5 and 7	Moral	Philosophy or ethics	United States	NA	NA	49
Tavani (2018)	Google Scholar: 1, 5, and 7, Scopus: 1, 5, and 7, and Web of Science: 1, 5, and 7	Rights/Moral	Philosophy or ethics	United States	4	Deontological	13
Theodorou (2020)	Google Scholar: 7	Moral	Computer engineering or computer science	Sweden	3	Other	0
Toivakainen (2016)	Google Scholar: 5 and 7 and ACM Digital Library: 5 and 7	Moral	Philosophy or ethics	Finland	NA	NA	3
Toivakainen (2018)	Google Scholar: 1 and Scopus: 1	Rights	Philosophy or ethics	Finland	2	Other	0
Tollon (2019)	Google Scholar: 5 and 7	Moral	Other humanities/Other social sciences	South Africa	4	Unclear	0
Tollon (2020)	Google Scholar: 7, Scopus: 5 and 7,and Web of Science: 5 and 7	Moral	Philosophy or ethics	South Africa	4	Social-relational	0
Tomasik (2011)	Google Scholar: 8	Suffering	Other or unidentifiable	Germany	5	Consequentialist	10
Tomasik (2013)	Google Scholar: 8	Suffering	Other or unidentifiable	Germany	5	Consequentialist	0
Tomasik (2014)	Google Scholar: 3, 6, and 8	Rights/Moral/Suffering	Other or unidentifiable	Germany	5	Consequentialist	8
Tonkens (2012)	Google Scholar: 7, ACM Digital Library: 7, and Scopus: 1	Rights/Moral	Philosophy or ethics	United States	NA	Virtue ethicist	22
Torrance (2005)	Google Scholar: 5 and 7	Moral	Other social sciences	United Kingdom	4	Mixture (consequentialist, deontological)	5
Torrance (2006)	Google Scholar: 5	Moral	Other social sciences	United Kingdom	4	Mixture (consequentialist, deontological)	3
Torrance (2008)	Google Scholar: 5 and 7, ACM Digital Library: 5 and 7, and Scopus: 5	Moral	Cognitive science	United Kingdom	4	Mixture (consequentialist, deontological)	89
Torrance (2011)	Google Scholar: 2	Rights	Other engineering/Computer engineering or computer science	United Kingdom	NA	NA	23
Torrance (2013)	Google Scholar: 2, 6, and 6	Rights/Moral	Other engineering/Computer engineering or computer science	United Kingdom	4	Mixture (consequentialist, deontological)	16
Torrance (2014)	Google Scholar: 6 and Scopus: 7	Moral	Other engineering/information technology	United Kingdom	4	Mixture (consequentialist, deontological)	19
Torres (2018)	Google Scholar: 10, ScienceDirect: 10, Scopus: 10, and Web of Science: 10	Suffering	Other or unidentifiable	United States	4	NA	15
Torres (2020)	Google Scholar: 9	Suffering	Philosophy or ethics	United States	4	Mixture (consequentialist, deontological)	0
Turchin (2019)	Google Scholar: 9	Suffering	Other or unidentifiable	United States	4	NA	0
Turchin et al. (2019)	Google Scholar: 9 and 10	Suffering	Other or unidentifiable	Russia	4	NA	5
Turner (2019)	Google Scholar: 1 and 7	Rights/Moral	Law	United Kingdom	3.5	Mixture (consequentialist, virtue ethicist, deontological, legal perspective)	70
Tzafestas (2016)	Google Scholar: 2	Rights	Other engineering	Greece	NA	NA	1
Umbrello and Sorgner (2019)	Google Scholar: 10	Suffering	Philosophy or ethics	Italy	4	NA	0
Vadymovych (2017)	Google Scholar: 1	Rights	Law	Ukraine	4.5	NA	3
Van den Berg (2011)	Google Scholar: 2	Rights	Law	Netherlands	NA	Legal precedent	13
Van den Hoven Van Genderen (2018)	Google Scholar: 2	Rights	Law	Netherlands	3.5	Legal precedent	2
Vanman and Kappas (2019)	Google Scholar: 5	Moral	Psychology	Australia	NA	NA	3
Vize (2011)	Google Scholar: 2 and 5	Rights/Moral	Philosophy or ethics	Australia	4	Consequentialist	0
Voiculescu (2020)	Google Scholar: 1 and 2	Rights	Other or unidentifiable	Unclear	NA	Legal precedent	0
Wallkötter et al. (2020)	Google Scholar: 5, 6, and 7, ACM Digital Library: 5 and 7, and Scopus: 5	Moral	Information technology	Sweden	NA	NA	0
Wang and Krumhuber (2018)	Google Scholar: 5 and 7	Moral	Psychology	United Kingdom	NA	NA	11
Ward et al. (2013)	Google Scholar: 7	Moral	Psychology	United States	NA	NA	45
Wareham (2013)	ACM Digital Library: 5	Moral	Medical or biology	Italy	4	Deontological	11
Warwick (2010)	Google Scholar: 1, ACM Digital Library: 1, Scopus: 1, and Web of Science: 1	Rights	Other engineering	United Kingdom	4	NA	103
Warwick (2012)	Google Scholar: 1 and 5 and ACM Digital Library: 5	Rights/Moral	Other engineering	United Kingdom	4	NA	14
Waser (2012)	Google Scholar: 3	Rights	Other or unidentifiable	Unclear	4.5	Unclear	10
Wegloop and Vach (2020)	Google Scholar: 9	Suffering	Other or unidentifiable	Germany	2.5	NA	0
Weng et al. (2009)	Google Scholar: 1	Rights	Other or unidentifiable	China	NA	Legal precedent	93
Winsby (2013)	Google Scholar: 5, 7, and 8	Moral/Suffering	Philosophy or ethics	Canada	4	Mixture (consequentialist, deontological)	2
Wortham (2018)	Google Scholar: 7	Moral	Computer engineering or computer science	United Kingdom	NA	NA	6
Wright (2019)	Google Scholar: 1 and 2	Rights	Law	United States	3.5	Legal precedent	0
Wu (2012)	Google Scholar: 2	Rights	Law	United States	3.5	Legal precedent	138
Wurah (2017)	Google Scholar: 1 and 2, Scopus: 2, and Web of Science: 2	Rights	Law	Canada	3	Legal precedent	5
Yampolskiy (2013)	Google Scholar: 1 and 7, ACM Digital Library: 1, Scopus: 1, and Web of Science: 1	Rights/Moral	Computer engineering or computer science	United States	1	NA (focused on AI safety concerns)	97
Yampolskiy (2017)	Google Scholar: 3 and 9 and Web of Science: 3	Rights/Suffering	Computer engineering or computer science	United States	4.5	NA	11
Yanke (2020)	Google Scholar: 1	Rights	Philosophy or ethics	United States	4	Mixture (legal precedent, consequentialism)	0
Zenor (2018)	Google Scholar: 1 and 4	Rights	Communication or media	United States	NA	Legal precedent	1
Ziesche and Yampolskiy (2018)	Google Scholar: 8, 9, and 10	Suffering	Other engineering	Maldives	4	Consequentialist	3
Ziesche and Yampolskiy (2019)	Google Scholar: 8 and 9	Suffering	Other engineering	Maldives	4	Consequentialist	2

**Table 8:** Discipline analysis

Discipline (or, for the bottom three, search term category)	Count and citations					Percentages of total		
	Items, counted	Citation count	Citation count (outlier excluded)	Average citation count	Average citation count (outlier excluded)	Items, counted	Citation count	Citation count (outlier excluded)
Business	3	17	17	6	6	1	0	0
Cognitive science	10	300	300	30	30	3	4	5
Communication or media	28	740	740	26	26	10	9	12
Computer engineering or computer science	30	473	473	16	16	10	6	8
Design	5	27	27	5	5	2	0	0
English literature or language	3	0	0	0	0	1	0	0
Future studies	2	3	3	2	2	1	0	0
History	3	83	83	28	28	1	1	1
Information technology	5	86	86	17	17	2	1	1
Law	49	1002	1002	20	20	17	12	17
Medical or biology	6	111	111	19	19	2	1	2
Other engineering	12	200	200	17	17	4	2	3
Other humanities	8	14	14	2	2	3	0	0
Other social sciences	18	111	111	6	6	6	1	2
Other or unidentifiable	32	471	471	15	15	11	6	8
Philosophy or ethics	82	4614	2101	56	26	28	54	35
Political science	3	13	13	4	4	1	0	0
Psychology	20	495	495	25	25	7	6	8
Robotics	4	23	23	6	6	1	0	0
Total	294	8505	5992	29	20	100	100	100
“Rights” search terms	146	2938	2938	20	20	50	35	49
“Moral” search terms	171	4071	4071	24	24	58	48	68
“Suffering” search terms	25	2700	187	108	8	9	32	3

**Table 9:** Framework analysis

Framework	Count and citations					Percentages of total		
	Items, counted	Citation count	Citation count (outlier excluded)	Average citation count	Average citation count (outlier excluded)	Items, counted	Citation count	Citation count (outlier excluded)
Consequentialist	16	2612	99	163	7	5	31	2
Deontological	15	213	213	14	14	5	3	4
Information Ethics	5	1019	1019	204	204	2	12	17
Legal precedent	34	906	906	27	27	12	11	15
Mixture	52	892	892	17	17	18	10	15
NA	103	1641	1641	16	16	35	19	27
Other	15	311	311	21	21	5	4	5
Social-relational	23	606	606	26	26	8	7	10
Unclear	26	277	277	11	11	9	3	5
Virtue ethicist	5	28	28	6	6	2	0	0
Total	294	8505	5992	29	20	100	100	100

**Table 10:** Country analysis

Country	Count and citations					Percentages of total		
	Items, counted	Citation count	Citation count (outlier excluded)	Average citation count	Average citation count (outlier excluded)	Items, counted	Citation count	Citation count (outlier excluded)
Argentina	1	4	4	4	4	0	0	0
Australia	15	85	85	6	6	5	1	1
Austria	3	9	9	3	3	1	0	0
Brazil	1	0	0	0	0	0	0	0
Canada	4	20	20	5	5	1	0	0
China	5	99	99	20	20	2	1	2
Colombia	1	6	6	6	6	0	0	0
Croatia	1	0	0	0	0	0	0	0
Cyprus	1	0	0	0	0	0	0	0
Czecia	1	0	0	0	0	0	0	0
Denmark	6	47	47	8	8	2	1	1
Finland	5	64	64	13	13	2	1	1
France	1	0	0	0	0	0	0	0
Germany	13	76	76	6	6	4	1	1
Greece	1	1	1	1	1	0	0	0
Indonesia	1	1	1	1	1	0	0	0
Iran	2	0	0	0	0	1	0	0
Ireland	2	17	17	9	9	1	0	0
Israel	2	26	26	13	13	1	0	0
Italy	7	472	472	67	67	2	6	8
Japan	6	88	88	15	15	2	1	1
Korea	2	0	0	0	0	1	0	0
Kuwait	1	3	3	3	3	0	0	0
Lithuania	4	49	49	12	12	1	1	1
Malaysia	1	0	0	0	0	0	0	0
Maldives	2	5	5	3	3	1	0	0
Malta	1	0	0	0	0	0	0	0
Netherlands	21	524	524	25	25	7	6	9
New Zealand	1	0	0	0	0	0	0	0
Norway	1	2	2	2	2	0	0	0
Poland	3	8	8	3	3	1	0	0
Russia	2	5	5	3	3	1	0	0
Singapore	1	0	0	0	0	0	0	0
Slovakia	1	1	1	1	1	0	0	0
South Africa	3	0	0	0	0	1	0	0
Spain	3	10	10	3	3	1	0	0
Sweden	3	0	0	0	0	1	0	0
Switzerland	4	5	5	1	1	1	0	0
Taiwan	1	3	3	3	3	0	0	0
Ukraine	2	7	7	4	4	1	0	0
Unclear	5	11	11	2	2	2	0	0
United Kingdom	44	4366	1853	99	43	15	51	31
United States	107	2453	2453	23	23	36	29	41
Total	294	8505	5992	29	20	100	100	100

**Table 11:** Dates analysis

Date	Publications that year	Cumulative total of publications
1988	1	1
1989	0	1
1990	0	1
1991	0	1
1992	0	1
1993	0	1
1994	3	4
1995	0	4
1996	1	5
1997	0	5
1998	0	5
1999	1	6
2000	0	6
2001	2	8
2002	1	9
2003	2	11
2004	3	14
2005	6	20
2006	4	24
2007	5	29
2008	6	35
2009	5	40
2010	6	46
2011	9	55
2012	17	72
2013	13	85
2014	14	99
2015	12	111
2016	27	138
2017	17	155
2018	40	195
2019	50	245
2020	49	294

**Table 12:** Journals and publication

Journal or book (if applicable)	Number of identified items	Percentage of all items
Chapter in a book	38	13
Entry in a conference report	36	13
NA	35	12
Ethics and Information Technology	25	9
AI and Society	13	4
Philosophy and Technology	7	2
Science and Engineering Ethics	6	2
Self-authored book	6	2
ACM SIGCAS Computers and Society	5	2
arXiv preprint	4	1
Computer Law and Security Review	4	1
Futures	4	1
Cognition	3	1
International Journal of Social Robotics	3	1
Law, Innovation and Technology	3	1
Artificial Intelligence and Law	2	1
IEEE Technology and Society Magazine	2	1
Journal of Evolution and Technology	2	1
Journal of Experimental and Theoretical Artificial Intelligence	2	1
Journal of Futures Studies	2	1
Journal of Information, Communication and Ethics in Society	2	1
Minds and Machines	2	1
Paladyn, Journal of Behavioral Robotics	2	1
Philosophies	2	1
ACM Transactions on Human–Robot Interaction (THRI)	1	0
Arkansas Law Review	1	0
Artificial Intelligence: Reflections in Philosophy, Theology, and the Social Sciences	1	0
Artnodes	1	0
Autism Research	1	0
Big Data and Cognitive Computing	1	0
BioLaw Journal-Rivista di BioDiritto	1	0
Cambridge Quarterly of Healthcare Ethics	1	0
Canberra Law Review	1	0
Cardozo Arts and Entertainment Law Journal	1	0
Case Research Paper Series in Legal Studies	1	0
Cognitive Science	1	0
Communications of the ACM	1	0
Connection Science	1	0
Critical Asian Studies	1	0
Current Biology	1	0
Dialogue: The Interdisciplinary Journal of Popular Culture and Pedagogy	1	0
Early Education and Development	1	0
Educational Insights	1	0
Environmental Ethics	1	0
Ethical Theory and Moral Practice	1	0
Ethics Quarterly	1	0
European Journal of Law and Political Sciences	1	0
Frontiers in Psychology	1	0
Game Studies	1	0
Harvard Journal of Law and Technology	1	0
Human Rights Quarterly	1	0
Human Rights Review	1	0
i-lex Scienze Giuridiche, Scienze Cognitive e Intelligenza Artificiale Rivista	1	0
Idea. Studia nad strukturą i rozwojem pojęć filozoficznych	1	0
Informatica	1	0
Information	1	0
Interaction Studies	1	0
International and Comparative Law Quarterly	1	0
International Journal of Psychology	1	0
International Review of Information Ethics	1	0
Iride	1	0
Islam and Civilisational Renewal ICR Journal	1	0
Journal of Experimental Child Psychology	1	0
Journal of Future Robot Life Preprint	1	0
Journal of Future Studies	1	0
Journal of Health, Social and Environmental Issues	1	0
Journal of Information, Communication and Ethics in Society	1	0
Journal of Medicine and Philosophy	1	0
Journal of Moral Education	1	0
Journal of Sociotechnical Critique	1	0
Journal of Virtual Worlds Research	1	0
Kairos: Journal of Philosophy and Science	1	0
Knowledge Futures: Interdisciplinary	1	0
Kritikos	1	0
Lentera Hukum	1	0
Machine Medical Ethics	1	0
Midwest Studies in Philosophy	1	0
NeuroImage	1	0
New Ideas in Psychology	1	0
New Media and Society	1	0
New Waves in Philosophy of Technology	1	0
Nordic Journal of Commercial Law	1	0
Northwestern University Law Review	1	0
On the Cognitive, Ethical, and Scientific Dimensions of Artificial Intelligence	1	0
Pandora’s Box—The journal of the Justice and the Law Society of the University of Queensland	1	0
Perception	1	0
Philosophical Investigations	1	0
Postmodern Culture	1	0
Procedia Computer Science	1	0
Proceedings of the International Association for Computing and Philosophy	1	0
Psychological Ownership and Consumer Behavior	1	0
Psychological Science	1	0
Review of Philosophy and Psychology	1	0
Revista de Filosofia Aurora	1	0
Robotics	1	0
Savannah Law Review	1	0
Science Fiction Film and Television	1	0
Scientia Moralitas-International Journal of Multidisciplinary Research	1	0
Scientific Reports	1	0
Social and Personality Psychology Compass	1	0
Social Cognition	1	0
Social Epistemology	1	0
South African Journal of Philosophy	1	0
Studies in History and Philosophy of Science Part C: Studies in History and Philosophy of Biological and Biomedical Sciences	1	0
The Frontiers of Society, Science and Technology	1	0
The Journal of Philosophical–Theological Research	1	0
Topoi	1	0
Trends in Cognitive Sciences	1	0
Universe, Human Immortality and Future Human Evaluation	1	0
University of Pennsylvania Law Review	1	0
Utah Law Review	1	0
Virginia Journal of Law and Technology Association	1	0
Writing Identity: The Construction of National Identity in American Literature	1	0

## Footnotes

1 See, for example, the comments on Barsanti (2017).

2 Vakkuri and Abrahamsson (2018) use a systematic methodology to examine key words. However, only 83 academic papers are examined, with “rights” only being returned as a key word in two of the articles and “moral patiency” in another three.

3 See “Focus and terminology.” For examples of their use, see Küster and Swiderska (2020) and Coeckelbergh (2010b).

4 See, for example, Wallach et al. (2008). We recognize that some moral frameworks may see moral agency as an important criterion affecting moral consideration (see, for example, Wareham, 2013; Laukyte, 2017). However, this criterion seems less directly relevant and including it in this literature review would have substantially widened the scope. Evaluations of agency and patiency may be correlated, but artificial entities may be assigned high agency alongside low patiency (Akechi et al., 2018; Gray et al., 2007). Lee et al. (2019) found that manipulations of patiency significantly affected perceived agency but that the reverse was not true. Items that explicitly discuss both agency and moral consideration were included (e.g. Johnson and Miller, 2008; Laukyte, 2017).

5 There are many contributions to this topic in other, less formal formats, such as blog posts. Given the huge amount of such literature, we excluded such items to provide a more coherent, interpretable literature review. Other thresholds, such as expert authorship, seemed less promising.

6 Gellers 2020 has subsequently been published. Several reviewed books discuss this topic but do not include it as their sole focus (e.g. Bostrom, 2014; Gunkel, 2012).

7 If Bostrom (2014) is included, then only 29% of citations were of items produced in the US, compared to 51% in the UK.

8 Presumably, sentient subroutines (as discussed in Tomasik, 2011) would not have humanoid shape, though some sentient simulations could have a humanoid shape in their simulated environment.

9 In the identified results, these adjectives tended to be used alongside “artificial” (see, for example, San José et al., 2016), though this may reflect the search terms used in this literature review. These adjectives were not included in the search terms because initial exploration suggested that the vast majority of returned results were irrelevant to the focus of this literature review.

10 Effective altruism is the approach of using our time and resources to help others the most (Sentience Institute, 2020). On CLR’s affiliation with this community, see Center on Long-Term Risk (2020).

11 For summaries, see Tavani (2018), Gunkel (2018a), and Bennett and Daly (2020).

12 See the section on “Empirical research on attitudes towards the moral consideration of artificial entities.”

13 Torrance (2008), Rademeyer (2017), and Laukyte (2017) also look “a bit further into the future” than other scholars to explore possible causes and effects of granting artificial entities legal rights.
