# Correction to: The Moral Consideration of Artificial Entities: A Literature Review

**DOI:** 10.1007/s11948-022-00373-6

**Published:** 2022-03-08

**Authors:** Jamie Harris, Jacy Reese Anthis

**Affiliations:** 1 Sentience Institute, New York, USA; 2 Department of Sociology, University of Chicago, 1126 East 59th Street, Chicago, IL 60637 USA

## Correction to: Science and Engineering Ethics (2021) 27:53 https://doi.org/10.1007/s11948-021-00331-8

In the original article published online one of the references is incorrect, which seems to be preventing it from being indexed.

It reads:

Jacy Reese, Anthis Eze, Paez (2021) Moral circle expansion: A promising strategy to impact the far future. Futures 130102756–https://doi.org/10.1016/j.futures.2021.102756.

But it should read:

Anthis, J.R., & Paez, E. (2021). Moral circle expansion: A promising strategy to impact the far future. Futures 130102756. https://doi.org/10.1016/j.futures.2021.102756

The original article has been corrected.

